# Understanding the factors related to how East and Southeast Asian immigrant youth and families access mental health and substance use services: A scoping review

**DOI:** 10.1371/journal.pone.0304907

**Published:** 2024-07-15

**Authors:** Chloe Gao, Lianne L. Cho, Avneet Dhillon, Soyeon Kim, Kimberlyn McGrail, Michael R. Law, Nadiya Sunderji, Skye Barbic

**Affiliations:** 1 Department of Medicine, University of British Columbia, Vancouver, British Columbia, Canada; 2 Department of Psychiatry, University of British Columbia, Vancouver, British Columbia, Canada; 3 BC Mental Health and Substance Use Services Research Institute, Vancouver, British Columbia, Canada; 4 Department of Occupational Science and Occupational Therapy, University of British Columbia, Vancouver, British Columbia, Canada; 5 Department of Psychiatry, McMaster University, Hamilton, Canada; 6 Waypoint Research Institute, Waypoint Centre for Mental Healthcare, Penetanguishene, Canada; 7 School of Population and Public Health, University of British Columbia, Vancouver, British Columbia, Canada; 8 Department of Psychiatry, Faculty of Medicine, University of Toronto, Toronto, Ontario, Canada; Access Alliance Multicultural Health and Community Services: Access Alliance, CANADA

## Abstract

The objective of the review is to identify factors related to how East and Southeast Asian immigrant youth aged 12–24 and their families access mental health and substance use (MHSU) services. To address how East and Southeast Asian youth and their families access mental health and substance use services, a scoping review was conducted to identify studies in these databases: PubMed, MEDLINE (Ovid), EMBASE (Ovid), PsychINFO, CINAHL, and Sociology Collection. Qualitative content analysis was used to deductively identify themes and was guided by Bronfenbrenner’s Ecological Systems Theory, the process-person-context-time (PPCT) model, and the five dimensions of care accessibility (approachability, acceptability, availability and accommodation, appropriateness, affordability). Seventy-three studies met the inclusion criteria. The dimensions of healthcare accessibility shaped the following themes: 1) Acceptability; 2) Appropriateness; 3) Approachability; 4) Availability and Accommodation. Bronfenbrenner’s Ecological Systems Theory and the PPCT model informed the development of the following themes: 1) Immediate Environment/Proximal Processes (Familial Factors, Relationships with Peers; 2) Context (School-Based Services/Community Resources, Discrimination, Prevention, Virtual Care); 3) Person (Engagement in Services/Treatment/Research, Self-management); 4) Time (Immigration Status). The study suggests that there is a growing body of research (21 studies) focused on identifying acceptability factors, including Asian cultural values and the model minority stereotype impacting how East and Southeast Asian immigrant youth access MHSU services. This review also highlighted familial factors (16 studies), including family conflict, lack of MHSU literacy, reliance on family as support, and family-based interventions, as factors affecting how East and Southeast Asian immigrant youth access MHSU care. However, the study also highlighted a dearth of research examining how East and Southeast Asian youth with diverse identities access MHSU services. This review emphasizes the factors related to the access to MHSU services by East and Southeast Asian immigrant youth and families while providing insights that will improve cultural safety.

## 1. Introduction

Mental health and substance use (MHSU) services worldwide are often characterized as fragmented, under-resourced, and inadequate to meet the needs of youth [1–5]. For young people who identify as immigrant East Asian (i.e., Chinese, Japanese, Hong Kong, Mongolian, Korean, Tibetan and Taiwanese) [6] or Southeast Asian (i.e., Burmese, Cambodian, Filipino, Indonesian, Thai, and Vietnamese) [6] youth, the barriers to obtaining MHSU care are even more significant [7–12]. The challenge of providing effective services that meet the diverse needs of this ethnoculturally minoritized population does not lie in the lack of evidence-based treatments for MHSU disorders but rather in the unavailability of culturally safe access points [13–19]. As a result, there is a crisis of access and engagement for East and Southeast Asian immigrant youth ages 12–24 needing culturally safe, youth-centred MHSU services [10, 12, 14, 20–24]. This is not surprising considering it has long been documented that Asian Americans use mental health services less frequently than the general population, with only 34.1% of Asian Americans with probable mental disorders seeking treatment as compared to 41.1% of their counterparts [25, 26]. Such findings can also be seen in a Canadian context; Canadian data has shown that Chinese British Columbians were less likely than other British Columbians to have reached out to a mental healthcare provider [16].

To address this crisis of access and engagement and deliver effective and culturally safe MHSU care, it is crucial to understand the cultural, social, and historical factors that influence East and Southeast Asian youths’ experiences of MHSU and their help-seeking behaviours in the existing literature [27–32]. Tailoring MHSU service delivery to specific populations while acknowledging their unique needs promotes effective and culturally safe care that leads to positive experiences [33–35]. Furthermore, engaging youth in MHSU research is particularly critical in the youth mental health sector [32, 36]. Involving youth in research can be therapeutic in itself by increasing confidence and developing new skills [32, 36]. In addition, youth engagement in MHSU research provides opportunities to connect with peers and draw on peer support networks while also developing skills that they can also apply to provide input into services [37]. Hence, youth-engaged MHSU research that supports the design, delivery, and evaluation of MHSU services can render services more responsive to youth needs, which, in turn, may improve service accessibility [37, 38]. In other words, youth can help organizations become more youth-friendly while broadening their own opportunities and improving their chances of success in various aspects of life [37, 38].

The crisis in MHSU access and engagement by Asian immigrants is likely driven, at least in part, by stereotypes [39]. The “model minority stereotype” (MMS) is the idea that certain minority groups, particularly Asian immigrants, are perceived as more successful, high-achieving, and well-adjusted compared to other minority groups [40, 41]. For this reason, Asian Americans were often compared to other racial minorities to perpetuate structural inequities during the civil rights movement [42]. A negative consequence of the MMS has been its infiltration into mental and physical health–that is, the perception that Asian immigrants in Western countries do not need research or clinical attention [43]. This has reduced resource allocation to this group in MHSU research, clinical care, and outreach [43, 44].

Another factor influencing MHSU access for this population lies in Asian immigrants having a stronger sense of collectivism than many other cultural groups [45]. Studies have linked the role of collectivism to an increased reliance on informal social networks to seek mental health support instead of venturing outside the family unit to seek more formalized support [46, 47]. This has been shown to contribute to a greater reluctance among Asian Americans to seek professional help compared to White Americans [30]. Family-oriented interdependence can also mean that any decision-making process, including the decision to obtain healthcare, is dictated by the interests of the family unit [45, 48]. As mental illness remains highly stigmatized in Asian immigrant communities where this topic is often taboo, concerns about revealing a perceived flaw to the community can spark shame for the whole family, which creates a barrier for accessing mental healthcare as youth with MHSU challenges may deny or hide their symptoms [48–50].

These barriers to accessing MHSU care for Asian immigrant youth are particularly concerning given the heightened risks they face of developing MHSU conditions [51]. For example, research highlights that many Asian immigrant young people grapple with the effects of stress and trauma experienced by their parents and previous generations [52–54]. For those who fled from areas with significant conflict, violence, war, or economic and political oppression, these experiences can often give rise to trauma that remains untreated due to the necessity of prioritizing physical survival [52]. Furthermore, the treatment of Asian immigrants in Western countries, such as the Chinese Head tax and Chinese Exclusion and Alien Land Acts in Canada and the US, respectively, as well as the World War II Japanese internment camps in both Canada and the US, is also a source of historical trauma that is often suppressed by Asian immigrant families [52, 55]. However, a comprehensive synthesis of potentially effective approaches to help Asian immigrant families communicate and process intergenerational trauma in culturally-safe ways has not yet been conducted [55–57].

Finally, it has long been documented that the Asian diaspora in Western countries faces structural racism and racial discrimination, with the COVID-19 pandemic causing a rise in anti-Asian hate crimes fuelled by racist rhetoric [58, 59]. Even before the deadly Atlanta-area spa shootings on March 16, 2021, which was both a race- and sex-based act of anti-Asian violence, Asian people have endured hate incidents in the community [60, 61]. Such racialized experiences may have direct implications for Asian immigrant youth adjustment, with a study implicating an association between discrimination and increases in anxiety, depressive symptoms, and sleep problems [62]. Despite this, there has been a dearth of research addressing the consequences of racism among East and Southeast Asian immigrant youth specifically [63].

It is vital that MHSU providers co-design and tailor MHSU services that consider the various factors and the current needs of youth to offer more effective and culturally safe care, considering this has been shown to reduce barriers to accessing treatment while promoting overall well-being [3, 37, 64]. To date, however, there has been no comprehensive overview of the individual-, familial-, community-, and system-level factors that influence East and Southeast Asian immigrant youths’ experiences of MHSU and their help-seeking behaviours. Therefore, we conducted a scoping review to elucidate how East and Southeast Asian immigrant youth and their families access MHSU services.

## 2. Methods

### 2.1. Definitions

In this review, a first-generation immigrant denotes the first of a generation to immigrate to any Western country (e.g., Canada, United States, New Zealand, United Kingdom, the Netherlands, France, Australia, etc.) [65–70]. First-generation immigrants also include refugees, referred to as individuals seeking asylum in another country, and undocumented, referred to here as individuals who do not possess a visa or any other form of immigration documentation [65, 71–73]. Second-generation immigrants are people born in Western countries with at least one first-generation immigrant parent [65–67]. Third-generation immigrants refer to people born in a Western country with two Western-born parents but at least one grandparent born outside of a Western country [65, 74, 75]. For this paper, first-, second-, and third-generation individuals will all be classified as “immigrants” and included in the study analysis [66, 71–73].

### 2.2. Guiding models

This review was informed by: 1) Bronfenbrenner’s Ecological Systems Theory, affording the application of ecology as a comprehensive theoretical approach embedding East and Southeast Asian immigrant youth within a larger social structure interacting with other social institutional structures; [76–78] 2) Bronfenbrenner’s process-person-context-time (PPCT) model [79–81] and 3) Five dimensions of healthcare accessibility, as conceptualized by Levesque and colleagues [82].

Bronfenbrenner’s Ecological Systems Theory involves five interconnected subsystems–the microsystem, mesosystem, exosystem, macrosystem, and chronosystem–that can be understood as an arrangement of nestled subsystems that extend from smallest to largest [76–78]. For this study, East and Southeast Asian immigrant youth are placed in the circle’s centre.

**Fig 1:**
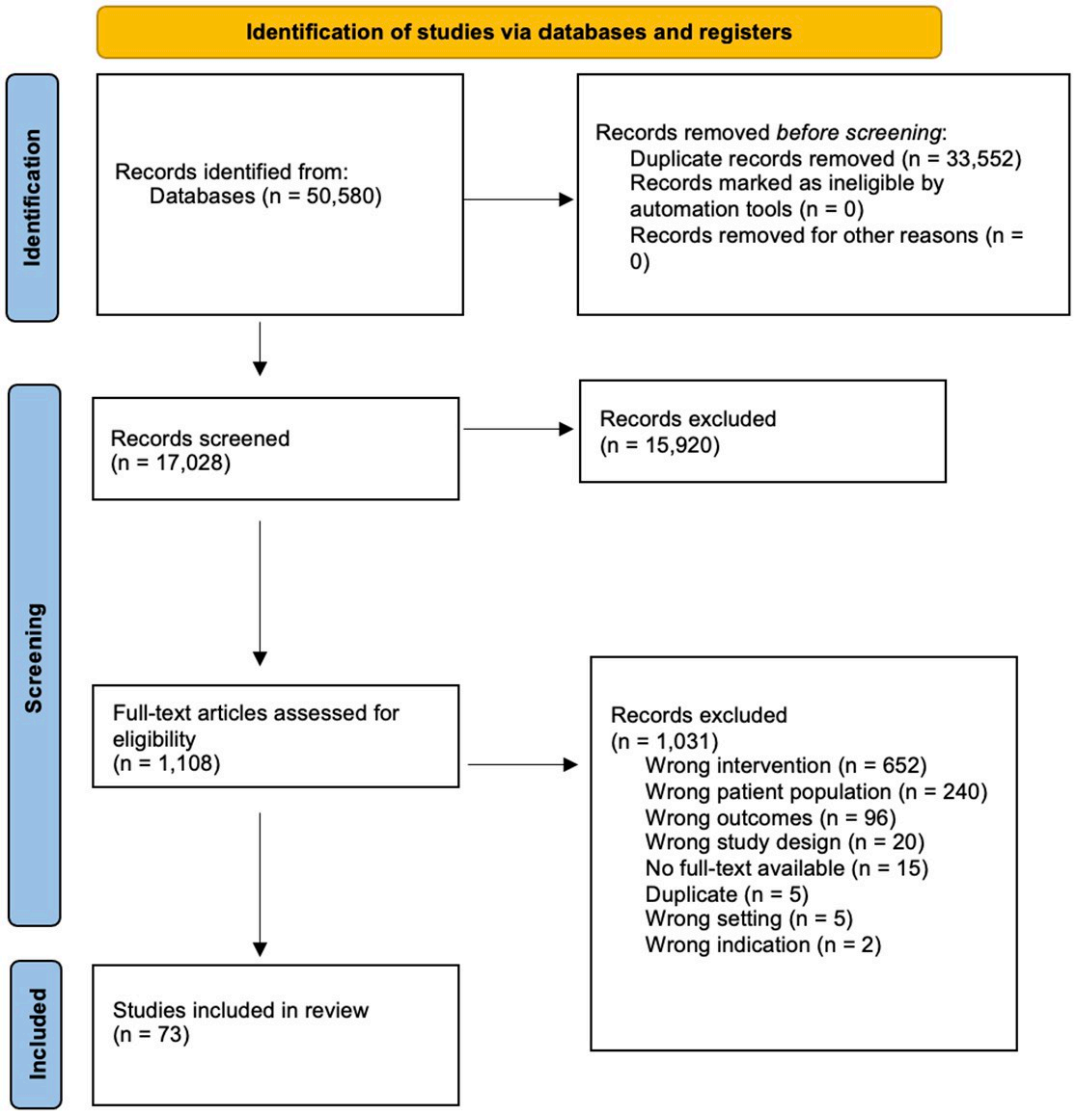
PPCT and Bronfenbrenner’s Ecological Systems Theory applied to how East and Southeast Asian immigrant youth access MHSU. Adapted from “Modeling Ecological Risk, Health Promotion, and Prevention Program Effects for Rural Adolescents,” by Q. Wu, 2019, *Journal of the Society for Social Work & Research*, *10*(1), 35. Copyright 2019 by the Society for Social Work and Research. Adapted with permission [86].

Bronfenbrenner later extended his ideas to develop the PPCT model [79, 80, 83]. In the PPCT model, proximal processes are characterized as the development of gradually more complex interactions and/or relationships between an individual and their surroundings [84, 85]. For personal processes, Bronfenbrenner postulates that an individual’s personal beliefs can reduce or increase the ability of proximal processes to impact individual behaviour and development [84]. Context refers to the environment, ranging from increasingly broad micro to macro levels [84]. Finally, time allows for examining the nature of intergenerational relationships, such as those between parents and children [84]. For this review, key themes were extracted from the literature and analyzed according to the four dimensions of the PPCT model (Fig 1) [79, 80, 83, 84].

Accessibility is a crucial aspect of healthcare systems, but accessibility has been defined in a heterogeneous manner in the literature [82]. In this work, we selected Levesque et al.’s conceptualization of access as it adequately describes expansive factors that balance demand and supply elements while clearly operationalizing healthcare access [82]. The five dimensions of accessibility are: 1) Approachability (i.e., people who have health needs can recognize that services exist, can be reached, and influence individual health outcomes); 2) Acceptability (i.e., factors affecting people’s ability to accept services); 3) Availability and Accommodation (i.e., services can be obtained in a timely and physically accessible fashion); 4) Affordability (i.e., people’s financial capacity to access services); and 5) Appropriateness (i.e., timeliness, the ability of services to meet the needs of service users, attention invested in the evaluation of health problems and the development of a treatment plan, and service quality) [82]. For this review, we used the five dimensions of accessibility to guide the analyses of results [82].

We synthesized and analyzed the patterns and means by which East and Southeast Asian immigrant youth access MHSU services to provide conceptual clarity (Table 1) [82].

**Table 1:** PCC criteria [89].

PCC Category	Description
**Population**	East and Southeast Asian immigrant youth and families (parents and/or guardians, or extended family of youth) [91–93]. Aged 12–24 for youth [94, 95].
**Concept**	Levesque et al. conceptualized access through five dimensions: 1) approachability; 2) availability; 3) affordability; 4) acceptability; and 5) appropriateness [82]. Bronfenbrenner’s Ecological Systems Theory and PPCT models will also guide analysis [79, 80, 83, 96].
**Context**	Research in any setting that captures the context of East and Southeast Asian immigrant youth seeking and/or accessing MHSU services. It is important to note that since barriers to access will also be explored, the population in question does not have to currently access MHSU services.

### 2.3. Review methods

We used a five-stage method outlined by Arksey and O’Malley (2002) to guide this scoping review [87]. The methods of this review were also guided by a scoping review conducted by Nesbitt et al. (2023) [88]. We did not complete a scoping review protocol a priori.

#### 2.3.1. Stage 1: Identifying the research question

We outlined our research question as: What is known about the individual-, familial-, community-, and system-level factors that influence how East and Southeast Asian immigrant youth and their families access MHSU services? We developed a research question in accordance with the population, concept, and context of interest (PCC), as the Joanna Briggs Institute Manual for Evidence Synthesis recommended [89, 90]. We defined each component a priori (Table 1).

#### 2.3.2. Stage 2: Identifying relevant studies

CG developed a search strategy in consultation with an experienced librarian at the University of British Columbia. CG searched the following online databases for study identification: MEDLINE (Ovid), EMBASE (Ovid), PubMed, PsychINFO, CINAHL, and Sociology Collection. The first literature search was conducted in July 2022, and then updated in May 2023 (see S3 for a full list of search strategies).

#### 2.3.3. Stage 3: Study selection

EndNote was used to remove duplicate articles for transferred to Covidence, a software for systematic reviews. CG then led the title/abstract screening and the subsequent full-text screening. In terms of eligibility assessment, studies were eligible for inclusion upon meeting the following criteria: a) Population: Referred to as East and Southeast Asian immigrant youth ages 12–24 and families seeking and/or accessing MHSU services (studies including participants beyond this age range were included if they overlap within this age range) [91–95]; b) Concept: Clarified the concept of access according to the five dimensions highlighted by Levesque et al. and/or discussed elements of Bronfenbrenner’s Ecological Systems Theory and PPCT models to provide insight into how East and Southeast Asian immigrant youth access MHSU [79, 80, 82, 83, 96]; c) Study type: All study types were considered except for review articles; and d) Language of publication and date: Written in English with no date restrictions.

#### 2.3.4. Stage 4: Charting the data

A data collection form was created and piloted by CG and SB to aid in data organization and interpretation. CG completed data extraction and SB checked the data extraction forms. The following participant characteristics were extracted: 1) the average age of study participants or age range of study participants (depending on how the study reported ages); 2) diagnoses (if present); 3) demographic information; and 4) sample size. Study details extracted were 1) the study purpose and research questions/objectives; and 2) study design (e.g., methods, service characteristics (if applicable), whether youth engagement was present). CG extracted qualitative themes that were highlighted and associated with MHSU service accessibility [79, 80, 82, 83, 96]. Since this is a scoping review and not a systematic review, a quality assessment was not conducted. However, study quality was considered when reviewing the evidence and triangulating different study results [97, 98].

One reviewer (CG) completed the extraction for all studies. Uncertainties in coding and interpretation throughout this review stage were discussed with the senior author, SB.

#### 2.3.5. Stage 5: Collating, summarizing, and reporting the results

We conducted qualitative content analysis to delineate patterns and factors influencing how East and Southeast Asian immigrant youth access MHSU [99, 100]. Qualitative themes emerged through inductive analysis through open coding and were subsequently grouped into categories in a deductive manner [79, 80, 82, 83, 96]. The coding and abstraction process was iterative; therefore, several variables extracted were then reorganized considering evolving understandings and narrative patterns, and the development of new subheadings. All content analyses were conducted by one reviewer (CG). All authors then collaborated to gradually refine the analyses and findings through several team-oriented iterative discussions.

We collated and reported our review findings according to the PRISMA-ScR Checklist [101]. In addition, we created an audit trail to keep track of noteworthy decisions among the authors of this review [97]. It has been widely noted that increasing the reflexivity of research can improve the credibility of qualitative data [102–106]. Therefore, guided by principles of reflexivity, we aimed to recognize how our lived experiences may colour our assumptions and biases about how East and Southeast Asian immigrant youth access MHSU services, which may impact our analyses of the collected data [107].

## 3. Results

**Fig 2:**
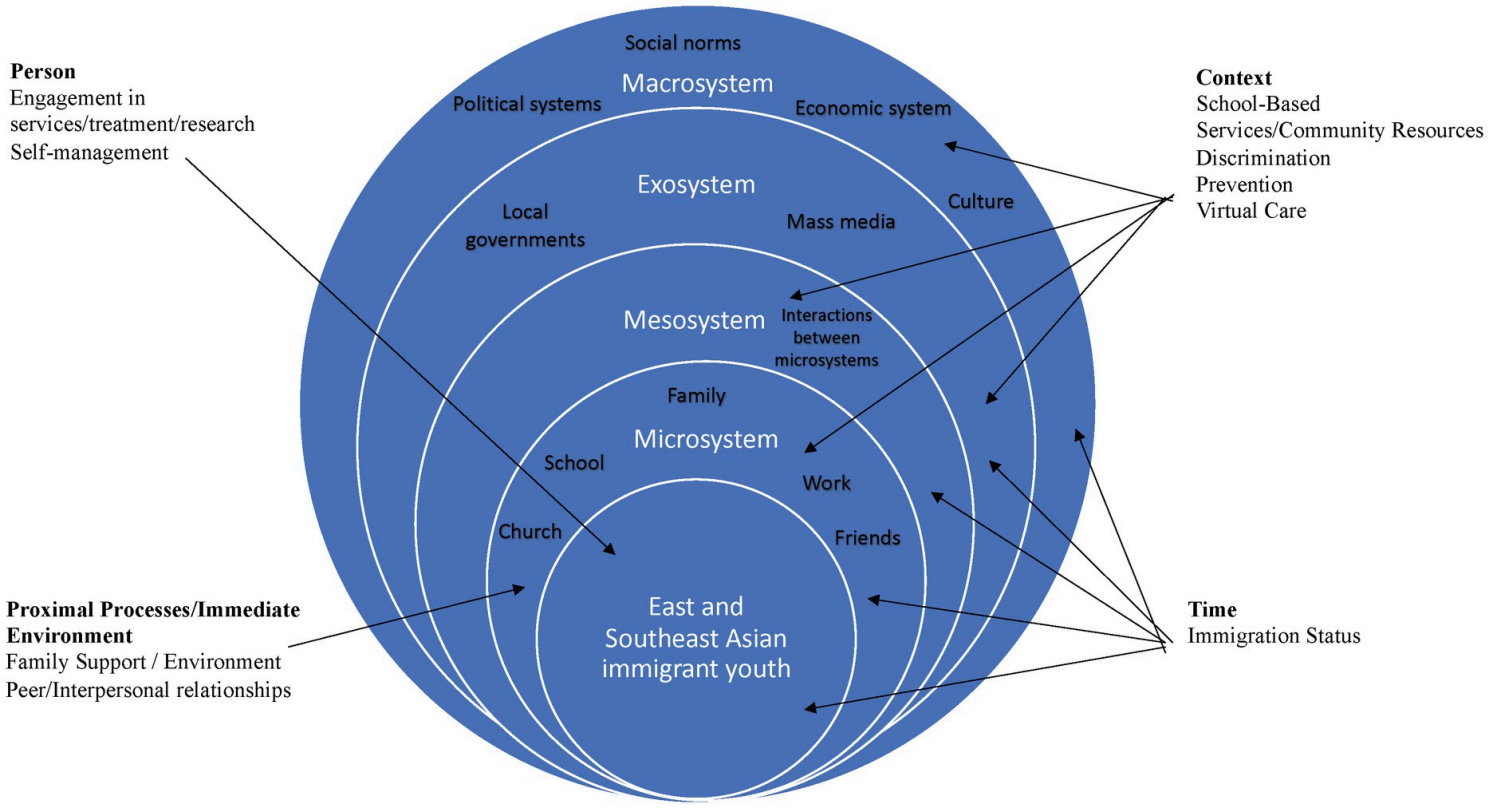
Flow diagram based on PRISMA guidelines for scoping reviews [101].

The initial search identified 50,580 records representing 17,028 unique articles. Screening titles and abstracts left 1,108 articles eligible for review against inclusion criteria. In total, 73 studies were assessed as eligible for inclusion (Fig 2).

The 73 studies analyzed in this review were published from 1977 to 2023. These studies were conducted in five countries: United States (n = 68), Canada (n = 1), United Kingdom (n = 1), New Zealand (n = 2), and Netherlands (n = 1). Characteristics of studies are outlined in Table 2 in S1 Table.

Across 73 studies, there was a total sample size of 9,504,599 participants. These studies often included participants aged 18 years and older rather than focusing solely on youth aged 12–24. The ages of participants in the included studies ranged from a mean of 7.8 (2.1 SD) years (childhood) [108] to no upper limit on the age range due to the inclusion of both adults 18–65 as well as parents/guardians of young people in this review. Samples were predominantly Asian, but 21 studies (28.8%) involved more diverse samples due to the addition of various racial and/or ethnic comparator groups (e.g., Indigenous, White, Latino, Black).

In terms of study design, 35 studies were cross-sectional surveys (47.9%), 17 studies were qualitative (23.3%), nine studies used mixed methods (12.3%), six studies were quantitative pre-post interventions (8.2%), four studies were retrospective cohorts (5.5%), two were case studies (2.7%), one study was a case-control (1.4%), and one study was both pre-post and qualitative in its design (1.4%). Thirty-six studies included a sex and/or gender-based analysis in the results (49.3%); however, 33 of the 36 studies (91.7%) did not clearly convey the difference between gender identity and biological sex by using sex and gender interchangeably and/or not specifically acknowledging the presence of more than two genders. Surprisingly, none of the studies captured sexual orientation in its demographic data collection. Furthermore, 23 of the 73 studies (31.5%) did not clarify the immigration status of participants, while 50 studies provided this information in various ways (e.g., first-/second-/or third-generation, or US-born/foreign-born/undocumented immigrant). Importantly, only six studies (8.2%) noted that they engaged youth in the co-creation of the research process.

The research question guiding this review was: What is known about the individual-, familial-, community-, and system-level factors that influence how East and Southeast Asian immigrant youth and their families access MHSU services? Guided by Bronfenbrenner’s Ecological Systems Theory, the PPCT model [76, 79, 84], and the five dimensions of healthcare accessibility [82], themes were deductively developed. The five dimensions of healthcare accessibility shaped the following themes: 1) Accessibility (Acceptability, Appropriateness, Approachability, Availability and Accommodation). One of the five dimensions of accessibility, affordability, did not have any relevant included studies. Bronfenbrenner’s Ecological Systems Theory and the PPCT model shaped the remainder of the themes: 2) Immediate Environment/Proximal Processes (Familial Factors, Relationships with Peers; 3) Context (School-Based Services/Community Resources, Discrimination, Prevention, Virtual Care); 4) Person (Engagement in Services/Treatment/Research, Self-management); 5) Time (Immigration Status) (Fig 1) [79, 80, 83, 96].

### 3.1. Accessibility

#### 3.1.1. Acceptability

Twenty-one studies discussed the dimension of acceptability in relation to how Asian immigrant youth access MHSU services [109–128]. Several studies found that Asian cultural values can give rise to public stigma, stigma by close others (e.g., family or friends), and self-stigma, which decrease acceptability of MHSU service use [109, 118–122, 125, 127, 129]. This may be explained by unique Asian cultural values such that being ‘different’ and obtaining mental health support stands in stark contrast to these values [109]. Therefore, people who strongly believe in Asian cultural values might be apprehensive about diverging from these norms and may avoid obtaining mental health support [109].

The literature also describes the MMS as a predictor of unfavourable help-seeking attitudes, which supports the view that the MMS has the potential to influence the perceived mental health functioning of Asian Americans [110, 111, 114]. As a result of the MMS, Asian Americans are perceived as mentally well irrespective of their mental health status [110, 111, 114]. However, Asian Americans’ convictions in environmental, biological and/or hereditary causes of mental health conditions increased their seeking professional mental health support [113, 126].

In terms of interventions to increase the acceptability of MHSU services among Asian immigrant youth, improving MHSU literacy has been cited as an important strategy [112, 113, 122–124]. Another commonly cited approach is implementing services whereby Asian immigrant youth are racially matched with their care providers, as perceived differences between themselves and their counselors in regards to worldviews and mental health beliefs is linked to less favourable ratings of the counselor, decreased willingness to visit the counselor, and less favourable counseling outcomes [115, 116, 128, 129].

#### 3.1.2. Appropriateness

Two studies described the dimension of appropriateness in relation to how Asian immigrant youth access MHSU services [129, 130]. Ngo-Metzger and colleagues showed that Asian Americans were less likely than White Americans to express that their physicians discussed lifestyle or mental health issues with them [130]. They were also more likely to express that their physicians did not understand their background and values [130]. When asked about the last visit, Asian Americans were more likely to mention that their doctors did not listen to them, spend an adequate amount of time providing care, or engage them in decision-making processes about their healthcare to the extent that they desired [130]. Such findings call into question concerns about the quality of services and user satisfaction [130].

Li and colleagues noted that a key challenge related to providing care for Asian American families is the lack of interpreter access, which renders services inappropriate for culturally and linguistically diverse families seeking care [129].

#### 3.1.3. Approachability

Four studies discussed the dimension of approachability about how Asian immigrant youth access MHSU care [131–134]. Included studies emphasized that Asian Americans are an underserved group compared to White Americans, with a lack of knowledge of where to seek help being cited as a barrier. Outreach interventions that normalize the positives and importance of seeking support may be an effective means of increasing service utilization among Asian Americans in need of mental health supports [131–134]. To increase favorable help-seeking outcomes among Asian Americans through outreach, Kim & Kendall (2015) suggest that assessing etiology beliefs may be effective in obtaining data related to an Asian American client’s help-seeking attitude [133]. For example, for a client who has firm spiritual or biological causes, the provider may posit that their help-seeking behaviours and intentions may be more favorable. Counselors may find it worthwhile to design psychoeducational outreach programs focusing specifically on the etiology of mental health problems, to facilitate help-seeking [133].

#### 3.1.4. Availability and accommodation

Three studies expand on the concept of availability and accommodation through various logistic difficulties, including language and communication difficulties in using a translator due to differences in language structure and emotional expressions [135], as well as documentation status serving as a barrier in accessing MHSU services among undocumented Asian and Pacific Islander young adults [136, 137].

### 3.2. Immediate Environment/Proximal Processes

#### 3.2.1. Familial factors

*3*.*2*.*1*.*1*. *Family conflict*. Four studies addressed the influence of family conflict on MHSU problems and help-seeking behaviours [137–140]. Family conflict was a common reason for seeking MHSU services [138]. Reasons for family conflict among Asian immigrant families included family tension due to immigration-related separation and reunification with different caretakers [137], cultural differences between children and parents [137], lack of time spent with children due to the multiple competing demands of parents struggling to support their families [137], and pressure by family to succeed academically and occupationally [139]. Individuals experiencing high levels of family conflict had a higher probability of seeking formal medical and mental health care services, suggesting that family conflict may act as a powerful interpersonal stressor, precipitating distress and subsequent help-seeking behaviours [137–140]. This demonstrates the importance of service providers in medical and MHSU sectors treating observations or reports of family conflict seriously, as such disclosure may indicate significant distress for Asian immigrants [138, 140]. It also suggests that certain MHSU interventions may be more appropriate for Asian immigrant youth and families, such as family and interpersonal therapies to address family conflict and relationship dynamics [141, 142].

*3*.*1*.*1*.*2*. *Reliance on family as support for help-seeking*. Two studies highlighted reliance on family as support [143, 144]. Lee (2015) emphasized how migration dissolved pre-existing social networks, precluding first-generation respondents from turning to local family networks for support [144]. In another study, both Asian American and Caucasian adolescents preferred to seek help from informal sources, such as parents and peers, rather than formal sources, such as therapists and school counselors [143]. Such findings emphasize the reliance on family for mental health support among Asian immigrants.

*3*.*1*.*1*.*3*. *Youth and parent MHSU literacy*. Two studies found that a lack of MHSU literacy was a commonly cited barrier to help-seeking [145, 146]. Asian immigrant parents often do not have an in-depth understanding of the causes and therapies for depression, with parents frequently neglecting their children’s depression until brought forward by schools [145]. Asian immigrant youth received parental perspectives that portrayed stigmatizing ideas of mental illness while negating its legitimacy and validity [146]. Parents often responded to young people experiencing distress by promoting culturally specific coping strategies, dismissing mental distress, or with no response [146]. Given that Arora & Khoo (2020) identified permission from parents to utilize mental health services and parental responses to expressed MHSU needs as being barriers to service access, these results emphasize the importance of promoting mental health literacy in Asian immigrant families [147].

*3*.*1*.*1*.*4*. *Culturally safe family-based interventions*. Eight studies highlighted culturally tailored family-based interventions for Asian immigrant youth and their families [108, 129, 148–153]. Highlighted interventions and their effects are outlined in Table 2.

**Table 2:** Culturally safe family-based interventions descriptions and effects.

Intervention(s)	Description	Effects
Ongoing community engagement related to knowledge translation of study findings [148].	Study findings were shared in a community conversation event focused on Filipino family wellness, multiple outreach presentations to encourage parents to enroll in programs aimed at improving parent-child relationships, and a toolkit on supporting mental health in the Filipino community [148].	No effects were reported.
Online substance use prevention program [149].	Nine-session virtual substance use prevention program for Asian American adolescent girls and mothers [149].	Intervention yielded increased closeness and communication between mothers and daughters, improved maternal oversight, and familial restrictions around substance use. Intervention also led to improvements in self-efficacy and refusal skills and decreased intentions to use substances. Intervention-arm girls engaged in decreased levels of alcohol and marijuana use and prescription drug misuse than control-arm counterparts [149].
Provider education/recommendations about culturally safe communication strategies with Asian American youth and their families [129].	The following strategies are recommended when promoting MHSU services among Asian American families: 1) Highlight that treatment can help school performance and honor the importance of the family in the client’s life; 2) Realize that ‘‘yes” does not always mean yes, as Asian American families typically value harmony and hierarchy rather than showing disagreement. Consequently, they may agree with the recommendation of the treatment providers despite not intending to follow through on recommendations; and 3) Note that there are still several challenges related to working with Asian American families [129].	No effects were reported.
Essay competition called “Hear Me Out” on topics of cross-generational communication among Asian American families [122, 151].	An essay competition entitled “Hear Me Out” was planned. Essay subjects related to intergenerational familial communication among Asian American youth and families to present their essays. Young people shared what they wanted their parents to understand while parents shared what they wanted their children to understand [122, 151].	Young people and their families acknowledged that they have different values due to differences in past experiences [122, 151].
ParentTeen Connect Workshop Series emerging from “Hear Me Out” [122, 151].	The ParentTeen Connect workshop series seeks to increase understanding of mental health among Asian American youth, improve parents’ knowledge of mental health, and promote supportive parenting strategies to support child and family mental health [122, 151].	Program was well-received by parents as it supported their learning of supportive parenting skills [122, 151].
Parent-child empowerment workshops for Asian American families [108].	Workshops covered topics such as acculturative stress, mental health, intergenerational conflict, quality of parent-child relationship, interpersonal difficulties, and racism. Participants had improved psychosocial functioning post-workshops [108].	Parental feedback emphasized the effectiveness of a holistic approach including group psychoeducation and in-session interventions developed to improve communication between parents and children [108].
Korean Family Communications (KFC) Program manual for Korean American parents of teens [152].	This parental training program strives to improve mental health literacy, stigma, family communication, and help-seeking attitudes [152].	Initial findings show increased mental health literacy and help-seeking attitudes. The program was well-regarded by Korean-American parents [152].
Program recommendation of preventive parenting programs in faith settings [153].	Healthcare providers recommended implementing preventive parenting programs in faith settings as a community-centred and culturally safe strategy to prevent Filipino youth mental health inequities [153].	No effects were reported.

Various positive outcomes resulted from these interventions such as increased mental health literacy among youth and families, improved sentiments about seeking MHSU services, parent-child relationships, and parenting skills, as well as increased resilience to substance use [108, 129, 148–152]. However, Li and colleagues noted several challenges that often preclude families from engaging in such interventions, including low MHSU literacy and stigma and shame about MHSU [129].

#### 3.2.2. Relationships with peers

Three studies discussed the influence of relationships with peers on MHSU help-seeking behaviours among Asian immigrant youth [139, 143, 154]. One mixed-method study found that many Chinese American students who had not used formal school health services but acknowledged a physical or mental health concern and sought support for this concern from teachers and peers [154]. In contrast, another mixed-method study found that Asian American youth have a preference for seeking help through peer networks rather than more formal networks such as counselors [143]. In addition, as part of a qualitative study, youth suggested using Asian American peers with personal experiences with mental health treatment sharing their direct experiences to reduce stigma and normalize mental health concerns among Asian American immigrant youth. Such findings provide further support for the role of formalized peer support programs to increase mental health literacy and reduce stigma among youth [139].

### 3.3. Context

#### 3.3.1. School-Based services/community resources

School-based MHSU services and/or community resources for Asian immigrant youth and families were addressed by nine studies [122, 131, 137, 139, 154–158]. One study revealed that religious identity was significantly associated with increased self-esteem over time and reduced depressive symptoms for females, but not for males [155]. Religious identity and participation were each positive and significantly linked to positive affect and the presence of meaning in life for both males and females [155]. These findings emphasize the utility of further examining how a religious community member may play a role in health and well-being, particularly among Asian American adolescents [155]. Goodkind (2005) developed an intervention for Hmong refugees that has two components: 1) Learning Circles involving cultural interchange and individual learning experiences for Hmong individuals; and 2) an advocacy component engaging undergraduate students advocating for and providing advocacy skills to Hmong families to increase access to resources within their community [156]. Participants’ increased quality of life could be attributed to their increased satisfaction with this community-based advocacy and learning program [156].

The other seven studies described the role of school-based services in influencing Asian immigrant youth’s mental health and well-being and how services are accessed [122, 131, 137, 139, 154, 157, 158]. Several studies found that Asian immigrant youth, controlling for confounders, had significantly lower odds of using their school-based MHSU prevention program than Black or Latino youth [131, 154]. Potential barriers to access include the shame of not living up to the MMS [137], not feeling welcomed at their school health programs [154], misconceptions of school health programs (e.g., services exist only for academic issues), and lack of awareness of the existence of such programs [139]. For those who did access services, relationships with school health program staff and their ability to refer to relevant school health programs served as key factors influencing youths’ openness to engage in stigmatized services, such as contraception counselling or mental health therapy [131, 154]. Furthermore, initiating school-based to promote mental health awareness [157] and programs that address the intersection of traditional and American values were recommended to improve MHSU literacy and service access in school settings [158]. It was recommended that teachers and administrators should also strive to create a positive school environment by encouraging the development of supportive relationships among students, their peers, and their teachers [122].

#### 3.3.2. Discrimination

One qualitative study highlighted that the MMS, the “perpetual foreigner” stereotype (presents racial minority youth as the “other” in White-dominant spaces) [159], or assumptions that Asian youth were more vulnerable to bullying, contributes to worsened mental health outcomes among Asian American youth. There was an acknowledgement of the importance of directly confronting discriminatory behaviours, which moderated the effectiveness of mental health services. Consequently, healthcare professionals, community leaders, and educators expressed that Asian American young people and families should strive to challenge racial discrimination and dismantle mental health stigma [137].

#### 3.3.3. Prevention

Two studies discussed approaches for preventing the onset of MHSU problems among Asian immigrant youth [160, 161]. Wang et al. (2022) and Havewala et al. (2022) culturally adapted youth mental health first aid for Asian Americans and found participants’ mental health literacy and their confidence in using mental health first aid skills significantly increased after the training [160, 161]. Such findings suggest that culturally tailored youth mental health first aid may improve mental health literacy and improve Asian American adults’ ability to support youth [160, 161].

#### 3.3.4. Virtual care

The rates of help-seeking for youth mental health services are lower within the Chinese community because of the limited communication strategies to ensure that healthcare information is accessible to this population [162]. However, one study highlighted that developing culturally safe smartphone applications for a Chinese immigrant community may increase knowledge about youth mental health and the delivery of services and resources [162].

### 3.4. Person

#### 3.4.1. Engagement in services/treatment/research

Regarding service engagement, which was addressed by 16 studies, one study found that English-speaking Asians were 11% more likely than English-speaking White people to discontinue mental health services [163]. Another study identified several person-related barriers to engaging in services for Asian immigrant youth, including discomfort opening up with others, confidentiality, beliefs around the perceived effectiveness of mental health treatment, and lack of time to dedicate to mental health service use [147].

Many studies noted that East and Southeast Asian immigrant youth had significantly lower MHSU service utilization than White individuals or the general population [164–171]. Asian American subgroups groups have different MHSU problems and service access patterns. Substance use disorders were most frequently observed in Southeast Asians. However, Southeast Asians with substance use disorders did not use mental health services as frequently in comparison to their South Asian counterparts. Similarly, East Asians, when compared to South Asians, had decreased odds of using mental health supports for their substance use disorders [172].

Studies reported that Asian immigrant youth have increasingly significant MHSU needs, as evidenced by a greater increase in admissions among Asian American and Pacific Islanders than non-Asian American and Pacific Islanders from 2000 to 2012 for substance use in the US [173]. Higher needs were associated with higher odds of MHSU service use [174, 175], and those who had used services also exhibited higher odds of reporting unmet needs [175].

In terms of engagement in research, two studies emphasized that youth can actively engage in multiple stages of the research process [176, 177]. Collaborative partnerships with Asian immigrant youth, alongside adults in the community, can aid in the development of culturally appropriate instruments and provide useful outcomes for research and community advocacy endeavors [176, 177]. As was previously stated, youth engagement in MHSU research to supports the design, delivery, and evaluation of MHSU services can increase service accessibility by making services more youth-centred and culturally-specific [37, 38].

#### 3.4.2. Self-management

One study highlighted self-management strategies for coping with mental health problems among Asian immigrant youth. Healthy coping mechanisms include playing sports and spending time with friends and family. In addition, healthcare professionals, community leaders, and educators stated that gang activity, substance use, online gaming addiction, school misconduct, and absenteeism were deemed the most common maladaptive coping strategies used by young people [137].

### 3.5. Time

#### 3.5.1. Immigration status

Ten studies described how generational immigration status affected how Asian immigrants accessed MHSU services, with mixed findings [109, 114, 137, 140, 167, 170, 171, 174, 176, 178]. Three studies found that US-born individuals (i.e., second-and-higher-generation immigrants) were more likely than foreign-born individuals (i.e., first-generation immigrants) to use MHSU services [167, 170, 174]. Such differences reflected perceptions of their treatment experiences [167]. In one study, perceived helpfulness of care differed by immigration status: Asian Americans born in the US, especially those who are third-generation or later, perceived services to be more helpful. The finding that second-generation and first-generation Asian Americans differ from third-generation Americans in their patterns of service use, as well as their perceptions on how helpful care is, suggest that second-generation individuals are more like their immigrant parents in how they access mental health services than their third-generation children [167]. However, one study found that the use of MHSU services did not reflect service needs; instead, unmet mental health needs were highest among non-US-born Asian American young adults ages 18–24 years [178].

Two studies commented on the unique experiences of second-generation immigrants relative to first- and third-generation immigrants [137, 140]. Chang and colleagues (2013) found that second-generation Asian Americans with higher levels of familial cultural conflict were more likely to utilize mental health services compared to their third-generation counterparts [140]. The role that family conflict plays in driving an uptick in mental health utilization by second-generation Asian Americans, compared with third-generation or later, highlights the uniqueness of the second-generation. According to the dissonant perspective of acculturation, conflict is magnified when second-generation immigrants acculturate faster to American culture than their first-generation parents, which, in turn, may render second-generation Asian immigrants more likely to seek MHSU services [179, 180]. Ling and colleagues (2014) also found that second-generation Asian American adolescents grappled with parental conflicts with parents who have a different cultural background, while first-generation newly immigrated adolescents experienced cultural and linguistic challenges, which impacted their self-esteem and agency [137].

In terms of research engagement to shape health and public policies, one study by Wong et al. (2015) highlighted that second-generation or later youth would be more likely to participate in research than newer migrants [176]. Given the aforementioned association between increased youth engagement in MHSU research and improved service accessibility and youth-centredness [37, 38], it is important to increase MHSU research engagement among newer migrants. Finally, three studies did not identify immigration status as a significant factor impacting how MHSUs are accessed among Asian immigrants in the US [109, 114, 171].

## 4. Discussion

This scoping review serves as a synthesis of factors related to how East and Southeast Asian immigrant youth access MHSU services. Guided by Bronfenbrenner’s Ecological Systems Theory [83, 96] and the five dimensions of healthcare accessibility conceptualized by Levesque et al. [82], the following themes were developed: 1) Intermediate Environment/Proximal Processes; 2) Context; 3) Person; 4) Time (Immigration Status); and 5) Accessibility (Approachability, Availability/Accommodation, Acceptability, Appropriateness). These themes reveal the complex processes underlying how East and Southeast Asian immigrant youth access MHSU services and the need to develop an intersectional understanding of such processes.

The current review uncovered a broad scope encapsulating how East and Southeast Asian immigrant youth access MHSU services and the barriers they experience. The central role of familial support, as well as the importance of improving the accessibility of care through addressing cultural barriers to seeking care, were uniquely highlighted in the review findings [109, 129, 131, 137, 144, 148–150]. Identifying acceptability factors affecting how East and Southeast Asian immigrant youth access MHSU services was the focus of the greatest number of studies [109–128]. The acceptability [109–128] factors highlighted align with findings from previous immigrant MHSU research highlighting cultural and social factors and the lack of fit between services and patients’ needs, as key challenges to accessing MHSU services [45, 181–185]. Furthermore, within the context of accessibility, researchers have increasingly emphasized the importance making MHSU services more accessible to ethnoculturally minoritized youth [31, 186, 187].

There was also a substantial portion of literature dedicated to identifying family influences on how MHSU services are accessed among East and Southeast Asian immigrant youth [108, 122, 129, 137, 138, 140, 143–153]. The family factors identified here, including family conflict, lack of MHSU literacy, reliance on family as support, and culturally safe family-based interventions, largely overlap with the family factors that have been highlighted in the adult Asian MHSU literature due to an emphasis on collective cultural strengths (i.e., family values) [108, 122, 129, 137, 138, 140, 143–152, 181, 182, 188–192]. Results demonstrate a variety of means by which family factors can be leveraged to develop more culturally safe services for East and Southeast Asian immigrant youth and families seeking MHSU care [115–119, 143, 150, 151].

However, findings suggest it is important to note that the family factors listed above cannot be understood in isolation of individuals’ micro-environment, such as peer relationships and self-management [139, 143, 154], as well as contextual factors such as education, structural racism, prevention, and the digitalization of care that impact how East and Southeast Asian immigrant youth access MHSU services [131, 137, 139, 153, 154, 160–162]. Several studies considered community/environmental resources that involved the youth’s immediate environment (school, personal support networks, health service quality and outcomes) [139, 143, 154]. Moreover, there is currently a lack of evidence on East and Southeast Asian immigrant youth belonging to two-spirit, lesbian, gay, bisexual, transgender, queer and/or questioning, intersex, asexual, and additional sexual orientations and gender identities (2SLGBTQIA+) [193–199]. Improving our understanding of the intersectional experiences of diverse East and Southeast Asian youth would reveal important social and contextual factors that give rise to MHSU inequities [200–203].

The review highlighted that East and Southeast Asian immigrant youth experience challenges and barriers while seeking MHSU services, which emphasizes the value of developing culturally safe care early intervention MHSU services for this population [204–206]. Furthermore, future research is needed to examine how racist stereotypes, such as the MMS, can be deconstructed at both individual- and macro-levels to improve the experiences of youth accessing care [111, 133, 207, 208]. Importantly, more research is needed to examine the unique strengths inherent in family structures and how these can be leveraged to support MHSU service access and healthy child development (e.g., commitment to their children and instrumental support practices, close intergenerational relationships, familial resilience and growth mindset) [45, 150, 151, 209–212]. Importantly, the majority of included studies reflect systematic issues in data collection, including practices that lead to the grouping of unlike individuals (e.g., Chinese, Vietnamese, and Bangladeshi) together in data collection systems [213]. The lack of disaggregated data for subpopulations with unique ethnic, cultural, linguistic, and migration histories fuels stereotypes of Asian immigrants and makes it difficult to delineate the drivers and experiences of health disparities among these diverse groups [213, 214]. Finally, considering the importance of engaging people with lived experience in the design, conduct, and dissemination of research findings, more research that engages East and Southeast Asian immigrant youth and families is warranted to gain a better understanding of the factors that are most important to those seeking MHSU services [10, 215].

The results of this review extended prior research on Asian immigrant youth MHSU by identifying factors related to how MHSU services are accessed rather than the state of Asian immigrant MHSU and/or MHSU service access [173, 216–219]. Moreover, while there have been reviews of evidence related to mental health status and the influence of cultural and social factors on mental health among Asian immigrants, there has not yet been a review focused specifically on factors influencing how East and Southeast young people access MHSU services [10, 12, 34, 201, 220–229]. Furthermore, by identifying factors that influence how East and Southeast Asian immigrant youth access MHSU services, findings can inform the development of culturally-safe MHSU services for this population [37, 230, 231]. Consequently, findings are relevant and valuable for the design, development, and evaluation of MHSU interventions that may positively promote East and Southeast Asian youth’s healthy development [37, 230, 231].

### 4.1 Strengths and limitations

This review has several limitations. Firstly, we did not perform a formalized quality assessment of the included studies, despite it being optional in scoping reviews [87, 98, 232]. Second, variability in how the population (youth), race (East and Southeast Asian immigrants), and concept (MHSU services) have been defined rendered it challenging to draw specific conclusions about how East and Southeast Asian youth ages 12–24 specifically access MHSU services. Moreover, 68 of the 73 studies were US-based, which may decrease the generalizability of the results to other Western countries and contexts due to the unique characteristics of the American healthcare system, as well as the distinct socioeconomic and political contexts [233]. We also recognize that this review was limited to studies that included East Asian and Southeast Asian youth. Opportunity exists for future research to explore the needs of other Asian ethnic populations (e.g., South Asian).

Using recent guidelines for conducting transparent and high-quality scoping reviews is a strength of the current review [97, 101, 234]. Additionally, this review was conducted by an interdisciplinary team with backgrounds in medicine (CG) public health (KM, MRL), occupational therapy/rehabilitation sciences (AD, SB), and psychiatry (LC, SK, NS). The diverse perspectives of the review team enriched the analyses and interpretation of data.

### 4.2. Future research directions

Based on this review, we recommend four areas for future research about how East and Southeast Asian immigrant youth access MHSU services:

1. Research that engages youth, family, and community members is warranted to generate research and knowledge translation activities that reflect the needs of MHSU service users [10, 112, 150, 156, 215].
2. How racist stereotypes, such as the MMS, can be deconstructed at both individual- and macro-levels to promote help-seeking behaviours and increased resource allocation for East and Southeast Asian immigrant youth seeking MHSU services [111, 133, 207, 208].
3. Understanding unique strengths inherent in family structures and how these can be leveraged to support MHSU help-seeking behaviours and healthy child development (e.g., commitment to their children and instrumental support practices, close intergenerational relationships, parental resilience and growth mindset) [45, 150, 151, 209–212].
4. Improving quality infrastructure and biases on the part of researchers, healthcare providers, and the public health community through disaggregating data for diverse subpopulations within Asian immigrant communities [213, 214].

### 4.3. Future considerations for practice and policy

The findings from this study have significant policy and practice implications. Considering the underutilization of MHSU services among East and Southeast Asian immigrant youth, studying these results can promote MHSU services among this understudied population by informing efforts to create culturally safe services for youth and families [235–237]. Findings from this study point to the importance of addressing biases and stereotypes among key stakeholders in a young person’s life (e.g., teachers and healthcare providers) to ensure that MHSU issues can be appropriately identified, diagnosed, and managed for East and Southeast Asian immigrant youth [111, 133, 207, 208]. Interventions focused on MHSU literacy, delivered to youth and families in community settings such as schools, might also improve knowledge about and reduce stigma related to MHSU issues [122, 131, 137, 139, 145, 146, 154–158]. Since youth often prefer to seek help from peers, implementing MHSU peer support services for East and Southeast Asian immigrant youth might help to decrease stigma and promote help-seeking behaviours in this population [139, 143, 154]. Moreover, to be truly culturally safe, policy and programmatic changes need to make evidence-informed decisions based on disaggregated datasets that account for the diverse diasporic subjectivities across subpopulations of Asian immigrant youth [213, 214]. Finally, programs and policies need to be rigorously evaluated through patient-oriented research to ensure interventions reflect the needs of East and Southeast Asian immigrant youth [238, 239].

## 5. Conclusions

The crisis of access and engagement for East and Southeast Asian immigrant youth needing culturally safe, youth-centred MHSU services has been acknowledged among researchers and clinicians. Review findings generate meaningful insights into factors affecting how East and Southeast Asian immigrant youth access MSHU services. Further research is encouraged that adopts family-centred, youth- and family-engaged strengths-based perspectives to enhance our understanding of MHSU service access among East and Southeast Asian immigrant youth and their families.

## Supporting information

S1 Table Details of included studies (n = 73) exploring how East and Southeast Asian youth access MHSU services. (DOCX)

S1 Checklist PRISMA-ScR checklist. (DOCX)

S1 File Full electronic search strategies for all databases. (DOCX)
