# Supplementary material for: Understanding the factors related to how East and Southeast Asian immigrant youth and families access mental health and substance use services: A scoping review
Source: PLoS One. 2024 Jul 15;19(7):e0304907. doi: 10.1371/journal.pone.0304907 (PMC11249267; doi:10.1371/journal.pone.0304907)
Supplement: S1 Table — (DOCX) [file pone.0304907.s001.docx]

**Table 2. Details of included studies (n = 73) exploring the factors and outcomes related to the access of mental health and substance use services by East and Southeast Asian immigrant youth and families**

| **Author** | **Country** | **Participant Characteristics: MHSU-related factors, Age (Range, Mean ± SD), Sex/Gender (%), Race/Ethnicity (%)** | **Sample Size (N)** | **Study Design** | **Primary Study Purpose** |
| --- | --- | --- | --- | --- | --- |
| Abe-Kim et al. (2002) | United States | **MHSU-related factors:** help-seeking for emotional distress from formal and informal sources, level of need (stress, health status, and psychological distress), demographic characteristics.  **Age:** 18-65 years. **Sex/Gender:** 49.6% female.  **Race/Ethnicity:** 100% Chinese | 1,503 | Retrospective cohort | To examine longitudinal predictors of help seeking for emotional distress in a community sample of Chinese Americans. |
| Abe-Kim et al. (2007) | United States | **MHSU-related factors:** demographic characteristics, mental health service use, treating ratings, diagnosis.  **Age:** age at time of immigration (≤12, 13-17, 18-34, and ≥35 years). **Sex/Gender:** n/r**. Race/Ethnicity:** 100% Asian [Chinese (n = 600), Filipino (n = 508), Vietnamese (n = 520), and other Asian (n = 467)] | 2,095 | Cross-sectional survey | To examine rates of mental health–related service use (i.e., any, general medical, and specialty mental health services) as well as subjective satisfaction with and perceived helpfulness of care in a national sample of Asian Americans, with a particular focus on immigration-related factors. |
| Anyon et al. (2014) | United States | **MHSU-related factors:** self-reported depressive symptoms, substance use, externalizing behaviour at school, failing grades, truancy, and discrimination by school adults and peers.  **Age:** n/r. **Sex/Gender:** 54% female.  **Race/Ethnicity:** 58% Asian (Chinese, Filipino, Vietnamese, Asian Indian, and Other). | 8,466 | Cross-sectional survey | To measure the prevalence of student risk factors and protective factors by race and ethnicity and assess the engagement of Asian youth in prevention services. |
| Anyon et al. (2013) | United States | **Phase 1 - Survey:**  **MHSU-related factors:** personal problems, engaging in early sexual activity, or using drugs.  **Age:** n/r. **Sex/Gender:** 50% female**.**  **Race/Ethnicity:** 57% Asian (42% Chinese American and 15% other Asian ethnic groups)  Phase 2 – Focus Groups and Interviews:  **MHSU risk factors:** personal problems, engaging in early sexual activity, or using drugs.  **Age:** n/r. **Sex/Gender:** 50% female**.**  **Race/Ethnicity:** 100% Chinese American | **Phase 1 – Survey:** 1,744  **Phase 2 – Focus Groups and Interview:**  44 | Mixed method | To examine whether school contextual factors, such as referral practices and peer dynamics, contribute to Chinese American students’ underrepresentation in school health programs. |
| Aratani et al. (2015) | United States | **MHSU-related factors:** dual diagnoses, mood disorders, psychotic disorders, developmental disorders, anxiety disorders, personality disorders, substance use, behavioural disorders, others, unknown diagnosis.  **Age:** 13.44 years (3.889 SD) – English speaking Asians; 11.89 years ( 4.637 SD) – non-English speaking Asians. **Sex/Gender:** 43.1% females – English speaking Asians; 35.1% – non-English speaking Asians. **Race/Ethnicity:** 14.6% Asian (Cambodian, Chinese, Filipino, Indian, Japanese, Korean, Laotian, Vietnamese). | 59,218 | Case-control | To explore the role of English proficiency, ethnicity, and California’s threshold language policy in the rates of discontinuing mental health services among Asian-American children. |
| Arora & Algios (2018) | United States | **MHSU-related factors:** n/r.  **Age:** 16.636 years. **Sex/Gender:** 57.6% females. **Race/Ethnicity:** 100% Asian (3.0% Bangladeshi, 3.0% Burmese, 75.8% Chinese, 3.0% Indian, 3.0% Korean, 3.0% Malaysian, 6.1% Pakistani, 3.0% Taiwanese). | 33 | Qualitative focus groups | To obtain in-depth information from urban-residing, first- and second-generation Asian American immigrant youth regarding their perceptions of school-based mental health services, as well as their recommendations for how schools can seek to better engage and address the mental health needs of these students. |
| Arora & Khoo (2020) | United States | **MHSU-related factors:** n/r.  **Age:** 16.636 years. **Sex/Gender:** 57.6% females. **Race/Ethnicity:** 100% Asian (3.0% Bangladeshi, 3.0% Burmese, 75.8% Chinese, 3.0% Indian, 3.0% Korean, 3.0% Malaysian, 6.1% Pakistani, 3.0% Taiwanese). | 33 | Qualitative focus groups | To examine the perceptions of Asian immigrant-origin youth regarding sources of stress that may contribute to mental health concerns and barriers to mental health service use. |
| Brice et al. (2015) | United States | **MHSU-related factors:** social anxiety measures and impairment measures.  **Age:** 14.91 years. **Sex/Gender:** 49% females. **Race/Ethnicity:** 5.5% Asian/Indian subcontinent | 3,837 | Cross-sectional survey | To examine rates of self-reported social anxiety symptoms among Asian American adolescents in an ethnically diverse sample of public high school students. |
| Chang et al. (2013) | United States | **MHSU-related factors:** lifetime service use, family cultural conflict, family-cohesion, immigration-related characteristics, history of mental health diagnosis, perceived need for mental health treatment, and demographic characteristics.  **Age:** 32.05% of sample between 18-34 years. **Sex/Gender:** 51.71% females. **Race/Ethnicity:** 45.06% Asian. | 4,649 | Cross-sectional survey | To examine ethnic and generational differences between Latino and Asian Americans in family cultural conflict and family cohesion and to ascertain the contributions of immigration- and family-related factors (e.g., generation status, family cohesion, family cultural conflict) to the underutilization of mental health services among Asian and Latino Americans. |
| Chen et al. (2008) | Canada | **MHSU-related factors:** mental health visits to general practitioners and visits to psychiatrists.  **Age:** Under 25 years at landing – 14; 25 or older at landing – 43. **Sex/Gender:** Under 25 years at landing – 50% females; 25 or older at landing – 51.5% females. **Race/Ethnicity:** 100% Chinese. | 152,184 | Retrospective cohort | To examine the individual and contextual factors associated with mental health visits to general practitioners and psychiatrists by recent Chinese immigrants in British Columbia. |
| Cheng et al. (2017) | United States | **MHSU-related factors:** Attitudes Towards Asian Americans Scale, Colourblind Racial Attitudes Scale, perceived mental health functioning, memory recall task.  **Age:** 19.54 years (**±** 2.92). **Sex/Gender:** 73% females. **Race/Ethnicity:** 5% Asian American/Pacific Islander. | 425 | Cross-sectional survey | To investigate the association between the model minority stereotype and perceived mental health functioning of Asian Americans in a college student population in order to test the pervasiveness of the stereotype and its general effect on the public. |
| Chiang et al. (2022) | United States | **Study 1**  **MHSU-related factors:** service selection for serious emotional problem, decision to seek help for everyday emotional issues.  **Age:** grades 9-12. **Sex/Gender:** 49% females. **Race/Ethnicity:** 67.3% East Asian.  **Study 2**  **MHSU-related factors:** open-ended questions about barriers to treatment, perceived importance of mental health, and unmet needs for adolescents and parents.  **Age:** ages 14 and 19. **Sex/Gender:** n/r. **Race/Ethnicity:** 100% Asian (Chinese, Indian, Korean, Vietnamese). | **Study 1**  759  **Study 2**  24 (12 adolescents, 12 parents) | Mixed methods | Study 1 (quantitative study): to examine help-seeking preferences during times of distress for a sample of Asian American and Caucasian high school students. Study 2 (qualitative study): to explore the rationale for Asian American adolescents’ help-seeking behaviours from two focus group discussions. |
| Cho et al. (2014) | United States | **MHSU-related factors:** predisposing factors, enabling factors, need factors.  **Age:** 18-97. **Sex/Gender:** n/r. **Race/Ethnicity**: 45% Asian (Chinese, Filipino, Vietnamese). | 4,649 | Cross-sectional survey | To examine factors associated with mental health service use among Latino and Asian Americans. |
| Choi & Miller (2014) | United States | **MHSU-related factors:** self-reported adherence to Asian cultural values, self-reported adherence to European American cultural values, public stigma toward seeking counseling, self-stigma of seeking counseling, attitudes toward seeking professional help, willingness to seek counseling.  **Age:** 21.74 years (3.77 SD). **Sex/Gender:** 68.34% females. **Race/Ethnicity:** 100% Asian American or Pacific Islander; Chinese (90; 32.4%), Korean (54; 19.4%), Asian Indian (28; 10.1%), Taiwanese (22; 7.9%), Filipino (17; 6.1%), Vietnamese (14; 5.0%), Japanese (10; 3.6%), Pakistani (eight; 2.9%), Thai (three; 1.1%), Nepali (three; 1.1%), Burmese (two; .7%), Bangladeshi (one; .4%), Cambodian (one; .4%), Malaysian (one; .4%), and Singaporean (one; .4%). | 278 | Cross-sectional survey | To test four theoretically and empirically derived structural equation models of Asian, Asian American, and Pacific Islanders’ willingness to seek counseling. |
| Choi & Kim (2010) | United States | **MHSU-related factors:** diagnosis of mental disorder, use of complementary and alternative medicines and/or traditional/conventional mental health services, sociodemographic characteristics, years in the United States/nativity, health status, English-language proficiency, perceived frequency of discrimination based on race/ethnicity, gender, age, skin colour, or other reasons.  **Age** 26% between 18-29 years, 45.6% between 30-49 years, 28.3% over the age of 50. **Sex/Gender:** 49% females. **Race/Ethnicity:** 100% Asian (Vietnamese, Filipino, Chinese, all other Asian). | 2,095 | Cross-sectional survey | To examine prevalence and correlates of the use of complementary and alternative medicines and traditional/conventional mental health services for mental health problems. |
| Coffey et al. (2022) | United States | **MHSU-related factors:** Likert-type question that asked the participants to rate if mental health was a problem among Filipinos by choosing one of the following responses: Not a problem at all (1); Only a minor problem (2); Somewhat a problem (3); A major problem (4); The number one problem (5).  **Age** 5.4% between 18-24 years, 43.2% between 25-39 years, 37.8% between 40-64 years, and 13.5% between 65-79 years. **Sex/Gender:** 62.2% females. **Race/Ethnicity:** 100% Filipino. | 37 | Qualitative focus groups | To develop a shared community definition of Filipino American adolescent mental health to address this critical issue. |
| Collier et al. (2012) | United States | **MHSU-related factors:** n/r.  **Age** 21.1 years. **Sex/Gender:** 46% females. **Race/Ethnicity:** 100% Hmong. | 13 | Qualitative focus groups | To verify the mental health needs of Hmong living in a Midwest U.S. community in order to clarify the format, content, and feasibility of providing mental health services for Hmong in the future. |
| Davis & Kiang (2016) | United States | **MHSU-related factors:** religious identity, religious participation, self-esteem, depressive symptoms, positive and negative affect, meaning in life, gender, generational status.  **Age** 15.03 years (0.92 SD). **Sex/Gender:** 60% females **Race/Ethnicity:** 100% Asian (Chinese, South Asian, Hmong, panethnic, multiethnic, Korean, Montagnard, Filipino, Laotian, Thai, Japanese, and Vietnamese. | 180 | Retrospective cohort study | To explore longitudinal variation in and associations between religiosity or the implications of these changes for psychological well-being. |
| Fang & Schinke (2013) | United States | **MHSU-related factors:** mother-daughter, mother-daughter communication, maternal monitoring, family rules about substance used, depressive symptoms, body esteem, self-efficacy, refusal skills, peer substance use normative beliefs, substance use intention, 30-day substance use.  **Age:** 13.10 years (0.96 SD) – daughter’s age**;** 39.73 years (6.81 SD) – mother’s age. **Sex/Gender:** 100% females. **Race/Ethnicity:** 100% Asian. | 108 | Pre-post | To test a parent-child program with a sample of Asian American adolescent girls and their mothers and evaluate the program’s efficacy on decreasing girls’ substance use and modifying risk and protective factors at individual, family, and peer levels. |
| Gamst et al. (2003) | United States | **MHSU-related factors:** consumer satisfaction composite and global assessment of functioning.  **Age:** 32.1 years. **Sex/Gender:** 59.4% females. **Race/Ethnicity:** 100% Asian (Chinese, Vietnamese, Japanese, Korean, Filipino, Laotian, Cambodian, Pacific Islander, or other). | 96 | Cross-sectional survey | To investigate the effects of consumer-provider racial match on consumer service satisfaction and treatment outcomes. |
| Goodkind (2005) | United States | **MHSU-related factors:** psychological well-being, quality of life, access to resources, English proficiency, and knowledge for the U.S. citizenship exam. **Age:** 41 years. **Sex/Gender:** 92.9% females. **Race/Ethnicity:** 100% Hmong. | 28 | Mixed methods | To assess the effectiveness of a community-based advocacy and learning intervention for Hmong refugees. |
| Green et al. (2020) | United States | **MHSU-related factors:** diagnostic assessment, barriers to treatment, reasons for dropout, race/ethnicity, socio-demographic covariates, country-level covariates. **Age:** 18-29 years. **Sex/Gender:** n/r. **Race/Ethnicity:** 7.8% Asian. | 1,417 | Cross-sectional survey | To examine racial/ethnic differences in perceived need for mental health treatment, barriers to treatment receipt, and reasons for dropout. |
| Havewala et al. (2022) | United States | **MHSU-related factors:** demographics, mental health literacy scale, confidence in mental health first aid skills, parental attitudes toward psychological services inventory, satisfaction scale, youth mental health opinions quiz. **Age:** 47 years (8.98 SD). **Sex/Gender:** n/r. **Race/Ethnicity:** 100% Asian. | 35 | Pre-post | To evaluate the effects of the youth mental health first aid virtual training among Asian Americans. |
| Jang et al. (2019) | United States | **MHSU-related factors:** mental health service use, mental health status, ethnicity, demographic variables. **Age:** 42.8 years (17.1 SD). **Sex/Gender:** 55.2% females. **Race/Ethnicity:** 640 Chinese (24.5%), 574 Asian Indians (22%), 471 Koreans (18.1%), 513 Vietnamese (19.7%), 265 Filipinos (10.2%), and 146 individuals from other Asian groups (5.6%). | 2,609 | Cross-sectional survey | (1) To explore the status of mental health, mental health service use, and perceived unmet needs for mental health care in Asian Americans and (2) To identify factors that determine the use of mental health services and perceived unmet needs for mental health care. |
| Javier et al. (2014) | United States | **MHSU-related factors:** open-ended questions regarding unmet mental health needs and recommendations for mental health prevention among Filipino youth. **Age:** Among adolescents, 11 were ages 14 to 17 (46%) and 13 were ages 18 to 21 (54%). Among caregivers, one was between the ages of 22 and 39 (9%), eight were between the ages of 40 to 64 (73%), and two were between the ages of 65 to 79 (18%). Among providers, 60% were ages 22 to 39, 27% were ages 40 to 64, and 13% were ages 65 to 79.  **Sex/Gender:** The adolescent sample included 12 females and 12 males. The caregiver sample included three males (27%) and eight females (73%). The provider sample included six males (47%) and eight females (53%).  **Race/Ethnicity:** 100% Filipino. | **Phase 1:**  33  **Phase 2:**  18 | Qualitative focus groups | To identify intervention strategies for implementing mental health prevention programs among Filipino youth. |
| Javier et al. (2010) | United States | **MHSU-related factors:** depressive symptoms and use of counseling among adolescents with a clinically significant level of depressive symptoms. **Age:** 12-17 years (8.98 SD). **Sex/Gender:** 46.1% females for Filipino; 49% females for White. **Race/Ethnicity:** 4.9% Filipino. | 4,421 | Cross-sectional survey | To compare measures of depressive symptoms and use of counseling in the past year for Filipino versus non-Hispanic White adolescents in California. |
| Jeong et al. (2017) | United States | **MHSU-related factors:** knowledge and beliefs about depression among Korean American parents of adolescents. **Age:** 44.7 years (4.5 SD) (parents of youth aged 12-19). **Sex/Gender:** 71.4% females. **Race/Ethnicity:** 100% Korean. | 14 | Qualitative focus groups | To explore depression literacy among Korean American parents of adolescents ages 12-19 using a qualitative descriptive design. |
| Jeong et al. (2018) | United States | **MHSU-related factors:** depression literacy, depression stigma, and attitude toward use of mental health care services in Korean American parents. **Age:** 46.7 years (5.0 SD) (parents of youth aged 12-19). **Sex/Gender:** 74.8% females. **Race/Ethnicity:** 100% Korean. | 141 | Cross-sectional survey | To examine mediating and moderating effects of depression literacy on the relationship between depression stigma and attitude toward use of mental health care services in Korean American parents. |
| Kim & Zane (2016) | United States | **MHSU-related factors:** perceived severity of symptoms, perceived susceptibility to mental health problems, perceived benefits of treatment, and perceived barriers to treatment. **Age:** 19.8 years (1.9 SD). **Sex/Gender:** 71% females. **Race/Ethnicity:** 60.2% Asian. | 656 | Cross-sectional survey | To understand potential reasons why Asian Americans underutilize mental health services relative to White Americans. |
| Kim & Kendall (2015) | United States | **MHSU-related factors:** emotional self-control, professional help-seeking attitudes, willingness to see a counselor, biological and spiritual etiology beliefs. **Age:** 20.10 years (1.76 SD). **Sex/Gender:** 68.5% females. **Race/Ethnicity:** 100% Asian (Korean, Chinese, Japanese, Filipino, Vietnamese, Taiwanese, Indian, Cambodian, Indonesian, Laotian, Hmong, and Thai) | 232 | Cross-sectional survey | To identify correlates of Asian American professional help-seeking using a mediation model describing Asian American help-seeking among college students. |
| Kim & Lee (2014) | United States | **MHSU-related factors:** help-seeking attitudes, internalized model minority myth, Asian values. **Age:** n/r. **Sex/Gender:** 67.9% females. **Race/Ethnicity:** 100% Asian (Chinese, Korean, Filipino, Japanese, Vietnamese, Taiwanese, Indian, and Cambodian) | 106 | Cross-sectional survey | To examine cultural factors underlying help-seeking attitudes of Asian American college students. |
| Lee (2006) | United States | **MHSU-related factors:** acculturation, social support, cultural identity, and health/mental health status, mental health service use. **Age:** 43 years. **Sex/Gender:** 53.3% females. **Race/Ethnicity:** 100% Vietnamese | 520 | Mixed methods | To understand how Vietnamese Americans use formal mental health services. |
| Lee & Jang (2016) | United States | **MHSU-related factors:** depressive symptoms, health insurance, acculturation, and personal beliefs about depression. **Age:** 29.1 years (6.17 SD). **Sex/Gender:** 53.9% females. **Race/Ethnicity:** 100% Korean. | 205 | Cross-sectional survey | To explore the factors associated with Korean immigrants’ willingness to use mental health services. |
| Lee et al. (2009) | United States | **MHSU-related factors:** (1) whether mental health was a concern in young adults in their community; (2) if so, what type of mental health issues were considered as problems; (3) possible sources of stress that affect mental health; (4) whether people with problems seek professional help; (5) if not, what are potential barriers for receiving professional help such as counseling or treatment; and (6) suggestions on possible development of prevention/education program. **Age:** 18-30 years. **Sex/Gender:** 88.2% females. **Race/Ethnicity:** 100% Asian (Indian, Cambodian, Chinese, Indonesian, Korean, Taiwanese, Thai, Vietnamese. | 17 | Qualitative focus groups | To obtain and discuss in-depth information on mental health problems, including the status, barriers, and potential solutions in 1.5 and 2nd generation Asian American young adults. |
| Lee et al. (2011) | United States | **MHSU-related factors:** lifetime prevalence of DSM-IV psychiatric disorders and mental health service utilization among various ethnic and racial groups. **Age:** 18-65 years. **Sex/Gender:** 55.2% females for Asians. **Race/Ethnicity:** 4.4% Asian, including Chinese (n = 306), Japanese (n = 175), Korean (n = 131), and Taiwanese (N=36); Southeast Asian (n = 485), including Filipino (n = 223), Indonesian (n = 29), Malaysian (n = 11), Vietnamese (n = 101), Thai, Laotian, Cambodian, or Burmese (n = 82), and other Pacific Islander (n = 39); or South Asian (n = 298), including Indian, Afghan, and Pakistani (n = 251) and Iranian (n = 47). | 43,093 | Cross-sectional survey | To compare the prevalence and odds of mental health service utilization among people of Asian ancestry with lifetime DSM-IV mood, anxiety, alcohol, and drug use disorders with utilization by members of other racial and ethnic groups with similar disorders. |
| Lee et al. (2015) | United States | **MHSU-related factors:** DSM-IV diagnosis, mental health service use. **Age:** over 18 years. **Sex/Gender:** 55.34% females. **Race/Ethnicity:** 100% Asian (East Asians, Southeast Asians, South Asians). | 1,431 | Cross-sectional survey | To analyze the National Epidemiological Survey on Alcohol and Related Conditions data, 2001–2002 and compare the prevalence and odds of DSM-IV mood, anxiety, and substance use disorders and mental health service use across Asian American subethnic groups. |
| Li et al. (2013) | United States | **MHSU-related factors:** clinical high-risk for psychosis. **Age:** 15 years. **Sex/Gender:** 0% female. **Race/Ethnicity:** 100% Chinese | 1 | Clinical case study | (1) To critically review Asian cultural values and beliefs about mental illness, psychosis, while highlighting specific challenges that Asian American families encounter; (2) to provide a clinical case to illustrate these challenges and inform clinical practice when working with Asian youth at risk for psychosis and their families, and (3) to provide practical and easy-to-follow clinical strategies. |
| Ling et al. (2014) | United States | **MHSU-related factors:** unmet mental health needs and challenges facing Asian and Asian American adolescents in the New York City metropolitan area. **Age:** n/r. **Sex/Gender:** 75% females. **Race/Ethnicity:** 81% East Asian **(**Chinese and Korean). | 16 | Qualitative interviews | To explore the perceived mental health needs of urban Asian American adolescents and barriers to meeting their needs from the perspective of social service providers using an ecological framework. |
| Liu et al. (2015) | Netherlands | **MHSU-related factors:** experiences of Chinese with mental health problems. **Age:** 37 years. **Sex/Gender:** 70% females. **Race/Ethnicity:** 100% Chinese. | 23 | Qualitative interviews | To investigate the experiences of Chinese with mental health problems, to inform measures to make services more responsive to the needs of this group. |
| Liu et al. (2022) | United States | **MHSU-related factors:** eating disorders, depression, helpful strategies to address mental health challenges at school. **Age:** 16.92 years (2.45 SD). **Sex/Gender:** 79.17% females. **Race/Ethnicity:** 100% Asian (Chinese, non-Chinese Asian). | 24 | Qualitative interviews | To explore Asian American adolescents’ understanding of eating disorders and depression and what they perceive as helpful strategies to address mental health challenges at school. |
| Mallinckrodt et al. (2005) | United States | **MHSU-related factors:** Suinn-Lew Asian Self-Identity Acculturation Scale and Etiology Beliefs Inventory. **Age:** 21.99 years (4.62 SD). **Sex/Gender:** 52% females. **Race/Ethnicity:** 100% Asian American or Pacific Islander. | 93 | Cross-sectional survey | To test the hypothesis that Asian American college students with the highest levels of acculturation, compared with those with low acculturation to Western culture, would share more beliefs about the causes of mental health problems in common with causal beliefs of typical counselors at university counseling centers. |
| Marchand et al. (2010) | United States | **MHSU-related factors:** depressive symptoms. **Age:** 17.3 years (1.6 SD). **Sex/Gender:** 75% females. **Race/Ethnicity:** 22% Asian. | 167 | Pre-post | To test whether a brief indicated cognitive-behavioral depression prevention program produced similar effects for Asian American, Latino, and European American adolescents. |
| Masson et al. (2013) | United States | **MHSU-related factors:** Addiction Severity Index, past and present help seeking experiences, treatment history and perceptions of substance use treatment programs, help seeking by others in their social network, and perceptions of the role of family, culture, and language in influencing help seeking behaviors. **Age:** 18-60 years. **Sex/Gender:** 29% females. **Race/Ethnicity:** 100% Asian American or Pacific Islander. | 61 | Mixed methods | To examine motivations and barriers to substance abuse treatment entry and treatment continuation among Asian American and Pacific Islander (AAPI) substance users. |
| McGarity-Palmer et al. (2023) | United States | **MHSU-related factors:** psychological distress and unmet mental health needs, overall and by nativity status. **Age:** 18-44 years. **Sex/Gender:** 61% females. **Race/Ethnicity:** 100% Asian American and Native Hawaiian/Pacific Islander. | 3,508 | Cross-sectional survey | To describe the prevalence of psychological distress and unmet mental health needs among Asian/Asian American adults during the COVID-19 pandemic across various sociodemographic subgroups. |
| Ngo-Metzger, et al. (2004) | United States | **MHSU-related factors:** respondents’ healthcare experiences, satisfaction with care, trust in doctor, and changing doctor. **Age:** 18-65 years. **Sex/Gender:** 57% females. **Race/Ethnicity:** 14% Asian (Chinese, Filipino, Asian Indian, Japanese, Vietnamese, Korean, Other Asian, Do not know/refused) | 3,726 | Cross-sectional survey | To examine how Asian race/ethnicity affects patients’ health care experiences and satisfaction with care. |
| Omizo et al. (2008) | United States | **MHSU-related factors:** collective self-esteem (membership, private, public, importance to identity), cognitive flexibility, general self-efficacy, and attitudes toward seeking professional psychological help. **Age:** 16.77 years (0.97 SD). **Sex/Gender:** 58% females. **Race/Ethnicity:** 100% Asian (Filipino, multiethnic Asian, Japanese, Chinese, and Korean). | 112 | Cross-sectional survey | To examine the extent to which Asian American adolescents who were living in Hawaii adhered to Asian and European American cultural values in relation to mental health variables including collective self-esteem (membership, private, public, importance to identity), cognitive flexibility, general self-efficacy, and attitudes toward seeking professional psychological help. |
| Pak (2023) | United States | **MHSU-related factors:** mental health literacy and attitudes towards seeking services. **Age:** parents of second-generation children aged 12-25 years.  **Sex/Gender:** 86% females in Study 1; 90% females in Study 2. **Race/Ethnicity:** 100% Korean. | 17 | Qualitative interviews & pre-post | To describe two studies that aimed to culturally adapt two existing physical therapy programs for first-generation Korean American parents of youth between 12-25 years. |
| Park et al. (2022) | New Zealand | **MHSU-related factors:** barriers to child and adolescent mental health service access. **Age:** parents of children aged 18 years and under.  **Sex/Gender:** n/r. **Race/Ethnicity:** 100% Korean. | 17 | Qualitative interviews | To explore reasons for the low rates of access to child and adolescent mental health services by the Korean community in New Zealand. |
| Sahker et al. (2017) | United States | **MHSU-related factors:** substance use treatment admission trends. **Age:** 12-55 years.  **Sex/Gender:** 29.5% females for Asian and Pacific Islanders and 32.6% females for non-Asian and Pacific Islanders. **Race/Ethnicity**: 1.5% Asian and Pacific Islanders. | 9,074,338 | Cross-sectional surveys | To evaluate the overall AAPI treatment admissions from 2000 to 2012. |
| Sangalang et al. (2015) | United States | **MHSU-related factors:** health and safety, family and cultural variables, community environment variables. **Age:** survey – average 16 years; focus groups – 14-18 years. **Sex/Gender:** survey – 51% females; focus groups – 60% females. **Race/Ethnicity:** 100% Cambodians. | survey – 475  focus groups – 40 | Mixed methods | To develop a health survey with Cambodian American youth using community-based participatory research (and illustrate how youth can actively engage in research to inform change in health-related programs and policies). |
| Stern et al. (1990) | United Kingdom | **MHSU-related factors:** referrals to the Department of Child Psychiatry at the London Hospital. **Age:** 0-15+ years.  **Sex/Gender:** 41.2% females. **Race/Ethnicity**: 14.3% Asian. | 189 | Retrospective cohort study | To report on the pattern of referrals to the child psychiatry department at the London Hospital with particular reference to Asian children. |
| Sudhinaraset et al. (2017) | United States | **MHSU-related factors:** documentation status, health status and healthcare access, community resources, social support.  **Age:** 22.9 years (3.3 SD)  **Sex/Gender:** 50% females. **Race/Ethnicity**: 100% Asian and Pacific Islander. | 32 | Qualitative interviews | To assess the psychosocial needs and health status of Asian and Pacific Islander undocumented young adults. |
| Thikeo et al. (2015) | United States | **MHSU-related factors:** demographics, acculturation scale, professional psychological help.  **Age:** 39.63 years (12.56 SD).  **Sex/Gender:** 65.7% females. **Race/Ethnicity**: (70.4 %) respondents from the Laotian community and 32 (29.6 %) from the Cambodian community. | 66 | Cross-sectional survey | To explore the demographic and acculturation factors in relation to attitudes toward seeking psychological help among Cambodian and Laotian Americans. |
| Ting & Hwang (2009) | United States | **MHSU-related factors:** attitudes toward seeking mental health services, general distress, demographics, social support and conflict, acculturation, stigma tolerance.  **Age:** 21.09 years (3.97 SD).  **Sex/Gender:** 66.36% females. **Race/Ethnicity**: 100% Asian (Chinese, Vietnamese, Japanese, Taiwanese, Korean). | 107 | Cross-sectional survey | To determine whether SBM generalizes to Asian American college students, and to determine whether the inclusion of culture-related variables (e.g., level of acculturation and stigma tolerance) help improve understanding of help-seeking attitudes above and beyond the culture-general variables described in the SBM. |
| Wang et al. (2019) | United States | **MHSU-related factors:** challenges faced by East Asian American immigrant families.  **Age:** n/r.  **Sex/Gender:** 77.8% females for youth; 63.3% females for parents. **Race/Ethnicity**: 100% Asian. | 38 | Qualitative case study | To describe the data collection process using the “Hear Me Out” events (essay contest, award ceremony/workshop) to better understand the challenges faced by East Asian immigrant families and to identify possible solutions to promote youth development. |
| Wang et al. (2020a) | United States | **MHSU-related factors:** mental health literacy, stigma toward mental illness, perceived barriers toward help seeking at middle or high schools.  **Age:** 17.13 years (2.33 SD).  **Sex/Gender:** 81.8% females. **Race/Ethnicity**: 50% Asian (Chinese, Vietnamese, Japanese, Taiwanese, Korean), 5.4% Asian/Latinx biracial. | 55 | Mixed methods | To explore the mental health literacy, stigma toward mental illness, and perceived barriers toward help seeking at middle or high schools among adolescents. |
| Wang et al. (2020b) | United States | **MHSU-related factors:** parent-child dynamics.  **Age:** first implementation – 47.92 years (3.34 SD); second implementation – 45.24 years (6.21 SD); third implementation – 44.00 years (6.14 SD).  **Sex/Gender:** first implementation – 76.9% females; second implementation –90.5% females; third implementation – 74.1% females **Race/Ethnicity**: 100% Chinese. | 25 | Mixed methods | (1) To build a university-community partnership and gather stakeholders’ input, (2) to identify community needs using qualitative data from the Hear Me Out events (including the essay contest and panel discussion), (3) to develop an intervention based on the analysis of the qualitative data and from extant literature on parent training programs, and (4) to pilot test the new parenting program. |
| Wang et al. (2020c) | United States | **MHSU-related factors:** parental beliefs about helpful strategies for addressing youth mental illness.  **Age:** 46.05 years (4.2 SD).  **Sex/Gender:** 89.5% females. **Race/Ethnicity**: 100% Asian (Taiwanese, Filipino, Chinese). | 19 | Qualitative interviews | To explore Asian American parents’ beliefs about helpful strategies for addressing youth mental illness (e.g. depression and eating disorders) with special attention to school-based strategies. |
| Wang et al. (2022) | United States | **MHSU-related factors:** demographics, mental health literacy scale, confidence in mental health first aid skills, acceptability.  **Age:** sample 1 – 45.55 years (3.60 SD); sample 2 – 23.17 years (3.67 SD).  **Sex/Gender:** sample 1 – 29% females; sample 2 – 87.5% females. **Race/Ethnicity**: 100% Asian (Chinese, Korean, Singapore, Malaysia, Filipino, Indian, Vietnamese, Pakistani, Sri Lankan, Thai, Other Asian country). | **Sample 1 –**  31  **Sample 2 –**24 | Pre-post | To culturally adapt youth mental health first aid for Asian Americans (parents and youth workers) and use a pre-test/post-test design to evaluate its effectiveness. |
| Wong (2006) | United States | **MHSU-related factors:** demographic variables; acculturation; lifetime, past year, and current experiences with treatment or counseling; psychological distress (clinical assessment of need); problem severity (perceived, subjective need); sources of help sought; perceived helpfulness of sources of help sought; willingness to seek professional mental health help; sources and perceived level of information about mental health problems and services; perceived facilitators to seeking professional mental health help; perceived barriers to seeking professional mental health help; belief in the model minority myth; somatic complaints; confidentiality concerns.  **Age:** 19.7 years (1.4 SD).  **Sex/Gender:** 64% females.  **Race/Ethnicity:** 100% Asian**.** | 224 | Cross-sectional survey | To examine factors influencing the help-seeking process in Asian American youth. |
| Wong et al. (2010) | United States | **MHSU-related factors:** lay beliefs about depression, enculturation to Asian values, and their likelihood of seeking professional help for depression.  **Age:** 23.57 years (6.76 SD).  **Sex/Gender:** 69.9% females.  **Race/Ethnicity:** 100% Asian (Chinese, Indian, Vietnamese, Filipino, Taiwanese, Korean, other Asian). | 223 | Cross-sectional survey | To examine the relations among Asian Americans’ lay beliefs about depression, enculturation to Asian values, and their likelihood of seeking professional help for depression. |
| Wong et al. (2014) | United States | **MHSU-related factors:** psychological professional help seeking for suicide ideation, disclosure of suicide ideation to others for the purposes of seeking social support, advice from others to seek professional help, strength of suicide ideation, demographics.  **Age:** 23.78 years.  **Sex/Gender:** 65% females.  **Race/Ethnicity:** 6.22% Asian. | 1,045 | Cross-sectional survey | To examine professional psychological help seeking among White American and Asian American students from US colleges and universities who had seriously considered attempting suicide. |
| Wong et al. (2015) | New Zealand | **MHSU-related factors:** health issues of priority concern to young Asian New Zealanders.  **Age:** 18-24 years.  **Sex/Gender:** 46.7% females.  **Race/Ethnicity:** 100% Asian (Taiwanese, Sri Lankan, Chinese, Indian, or Malaysian Chinese ethnic groups) | 15 | Qualitative study | To identify young Asian New Zealanders’ perspectives on best approaches to investigate health issues of priority concern to them. |
| Wong et al. (2018) | United States | **MHSU-related factors** beliefs, attitudes, and intentions toward mental illnesses and treatment.  **Age:** 23% of sample between 18-24 years.  **Sex/Gender:** 64% females.  **Race/Ethnicity:** 8% Asian American (n = 327). | 4,122 | Pre-post | To examine whether the effects of contact-based educational programs varied depending on the age, gender, and race-ethnicity of participants. |
| Wu et al. (2018) | United States | **MHSU-related factors:** demographic characteristics, smartphone use patterns, interest level for learning child behaviour via smartphone apps among all participants.  **Age:** 35.8 years.  **Sex/Gender:** 79% females.  **Race/Ethnicity:** 100% Asian (Chinese, Indian, Vietnamese, Filipino, Taiwanese, Korean, other Asian). | 100 | Cross-sectional survey | To evaluate the feasibility of a customized smartphone application that can deliver child mental health resources effectively to Chinese immigrant parents. |
| Wu & Lee (2015) | United States | **MHSU-related factors:** demographic questionnaire, psychosocial outcome rating scale, children’s action tendency scale, revised children’s manifest anxiety scale-2, school performance measure.  **Age: starting at** 7.8 years (2.08 SD). **Sex/Gender:** 50% females for children; 58% females for parents.  **Race/Ethnicity:** 100% Asian (Chinese, Japanese, Korean, Vietnamese, Taiwanese, Other). | 24 | Mixed methods | To evaluate the perceived effectiveness of a brief, community-based parenting intervention for Asian immigrant families in the United States. |
| Yang et al. (2020) | United States | **MHSU-related factors:** past-year respondent-perceived need for mental health treatment, past-year serious psychological distress, past-year major depressive episode, treatment outcome, demographic/health-related covariates.  **Age:** 18-64 years.  **Sex/Gender:** 53.1% females for Whites; 52.2% females for Asians.  **Race/Ethnicity:** 7.49% Asian. | 116,525 | Cross-sectional survey | To examine mental health treatment access disparities between Asians and Whites in the United States as well as the role of perceived and objective need and barriers to treatment in these disparities. |
| Yasui et al. (2022) | United States | **MHSU-related factors** youth-reported parental socialization of mental health within Chinese American families.  **Age:** 18 years.  **Sex/Gender:** 53% females.  **Race/Ethnicity:** 100% Chinese. | 69 | Qualitative focus groups | To explore youth-reported parental socialization of mental health within Chinese American families by examining focus group data from Chinese American high school and college students. |
| Yee et al. (2020) | United States | **MHSU-related factors** youth-reported parental socialization of mental health within Chinese American families.  **Age:** 32.83 years (9.72 SD).  **Sex/Gender:** 60.7% females.  **Race/Ethnicity:** 100% Chinese. | 229 | Cross-sectional survey | To propose a mediation model to clarify the relationships amongst the variables impacting help-seeking attitudes. |
| Yee & Lee (1977) | United States | **MHSU-related factors**: mental health primary prevention program for Filipino youth.  **Age:** grades 10-12.  **Sex/Gender:** n/r.  **Race/Ethnicity:** 100% Filipino. | 45 | Qualitative program evaluation | To describe a school primary program for Asian American youth. |
| Yeh et al. (1994) | United States | **MHSU-related factors**: parallel or mainstream mental health centers, client characteristics, program characteristics, utilization, outcome.  **Age:** 12.76 years (4.24 SD) for mainstream; 11.94 years (4.25 SD) for parallel.  **Sex/Gender:** 51% females for mainstream; 44% females for parallel. **Race/Ethnicity:** 100% Asian. | 912 | Cross-sectional survey | To examine differences between parallel ethnic-specific and mainstream outpatient mental health services for Asian American children. |
| Zane & Cho (2011) | United States | **MHSU-related factors:** racial match, critical counselling processes (counselor credibility, working alliance, perceived similarity, perceived support, ethnic identity) **Age:** 18.94 years (1.29 SD). **Sex/Gender:** 78.9% females. **Race/Ethnicity:** 100% Asian (Chinese, Vietnamese, Korean, Japanese, Southeast Asian). | 171 | Cross-sectional survey | To understand the effect of racial match on critical counseling processes (i.e., therapist credibility and the working alliance) among Asian Americans. |
| Zhou et al. (2022) | United States | **MHSU-related factors**: acculturation status, internalization of thin and muscular body ideals, body surveillance, body shame, and disordered eating.  **Age:** 20.36 years (1.58 SD).  **Sex/Gender:** 66% females. **Race/Ethnicity:** 100% Asian (Chinese/Taiwanese, Vietnamese, Korean, Filipino, Japanese, South Asian, Indian, Nepalese, etc.). | 245 | Cross-sectional survey | To contribute to the limited existing research on acculturation and disordered eating among Asian American college students who represent an understudied and high-risk group. |

Abbreviations

**AAPI:** Asian American Pacific Islander

**DSM-IV:** Diagnostic and Statistical Manual of Mental Disorders

**MHSU:** Mental Health and Substance Use

**SBM:** Society of Behavioural Medicine

**SD:** Standard Deviation

**USA:** United States of America
