# Supplementary material for: Understanding the factors related to how East and Southeast Asian immigrant youth and families access mental health and substance use services: A scoping review
Source: PLoS One. 2024 Jul 15;19(7):e0304907. doi: 10.1371/journal.pone.0304907 (PMC11249267; doi:10.1371/journal.pone.0304907)
Supplement: S1 File — (DOCX) [file pone.0304907.s003.docx]

| 1 | exp Mental Disorders/ |
| --- | --- |
| 2 | ((mental or psychiatric) adj3 (illness or disorder* or disease*)).mp |
| 3 | (anxiety or depression or mental health) |
| 4 | 1 or 2 or 3 |
| 5 | asians/eh or asian americans/eh |
| 6 | exp china/eh or exp japan/eh or exp korea/eh or exp mongolia/eh or exp taiwan/eh |
| 7 | (East Asian or Korean or Hmong or Thai* or Lao* or Japan* or Vietnamese or Filipino or Chinese or Cambodian) |
| 8 | 5 or 6 or 7 |
| 9 | 4 and 8 |
| 10 | attitude to health / or health knowledge, attitudes, practice / or “treatment adherence and compliance”/ or “patient acceptance of health care”/ or patient compliance / or medication adherence / or no-show patients / or patient dropouts / or patient participation / or patient satisfaction / or patient preference / or treatment refusal / |
| 11 | (experience* or outcome* or quality or satisfaction or attitude* or preference* or participation or engagement) |
| 12 | exp narration / or exp narrative medicine / or exp patient-centred care / or exp interviews as topic / or exp personal narratives as topic / or exp biographies as topic / or exp autobiographies as topic / |
| 13 | (narration or “narrative medicine” or “patient-centred care” or interview or biography or autobiography or “personal narrative*” or “lived experience” or “patient narrative*” or “patient story”) |
| 14 | 10 or 11 or 12 or 13 |
| 15 | 9 and 14 |
| 16 | (adolescent* or young adult* or teen*) |
| 17 | 15 and 16 |

Database(s): **Ovid MEDLINE(R) and Epub Ahead of Print, In-Process, In-Data Review & Other Non-Indexed Citations, Daily and Versions(R)** 1946 to May 2023

| 1 | exp mental disease/ |
| --- | --- |
| 2 | ((mental or psychiatric) adj3 (illness or disorder* or disease*)).mp |
| 3 | (anxiety or depression or mental health) |
| 4 | 1 or 2 or 3 |
| 5 | east asian / or chinese / or “japanese (citizen)” / or “mongolian (citizen)” / or north korean / or south korean / or taiwanese / |
| 6 | (East Asian or Hmong or Lao* or Korean or Japan* or Vietnamese or Filipino or Chinese or Cambodian or Taiwan* or Mongolia* |
| 7 | asian american/ |
| 8 | 5 or 6 or 7 |
| 9 | 4 and 8 |
| 10 | attitude to health |
| 11 | patient compliance / or mental compliance / or patient attitude / or medication compliance / |
| 12 | patient dropout/ |
| 13 | patient participation/ |
| 14 | patient satisfaction/ or satisfaction/ |
| 15 | patient preference/ |
| 16 | treatment refusal |
| 17 | (experience* or satisfaction or attitude* or preference* or participation or engagement) |
| 18 | (narration or “narrative medicine” or “patient-centered care” or interview or biography or autobiography or “personal narrative*” or lived experience” or “patient narrative*” or “patient story”) |
| 19 | exp interview/ or exp narrative medicine/ |
| 20 | 10 or 11 or 12 or 13 or 14 or 15 or 16 or 17 or 18 or 19 |
| 21 | 9 and 20 |
| 22 | (adolescent* or young adult* or teen*) |
| 23 | 21 and 22 |

Database(s): **Embase and Epub Ahead of Print, In-Process, In-Data Review & Other Non-Indexed Citations, Daily and Versions(R)** 1974 to May 2023

Database(s): **PubMed** 2001 to 2023

[(((mental OR psychiatric) NEAR/3 (illness OR disorder* OR disease*)) OR (anxiety OR depression OR "mental health")) AND ("East Asian") AND ((experience* OR outcome* OR quality OR satisfaction OR attitude* OR preference* OR participation OR engagement) OR (narration OR "narrative medicine" OR "patient-centered care" OR interview OR biography OR autobiography) OR ("personal narrative" OR "personal narratives") OR ("lived experience" OR "patient narrative*" OR "patient story")) AND (adolescent* OR young adult* OR teen*)](https://www.proquest.com/myresearch/savedsearches.checkdbssearchlink:rerunsearch/2378884/SavedSearches?site=sociology1&t:ac=SavedSearches)

Database(s): **APA PsycInfo** 1887 – May 2023

| 1 | DE "Mental Disorders" OR DE "Affective Disorders" OR DE "Anxiety Disorders" OR DE "Autism Spectrum Disorders" OR DE "Bipolar Disorder" OR DE "Borderline States" OR DE "Chronic Mental Illness" OR DE "Dissociative Disorders" OR DE "Eating Disorders" OR DE "Gender Dysphoria" OR DE "Mental Disorders due to General Medical Conditions" OR DE "Neurocognitive Disorders" OR DE "Neurodevelopmental Disorders" OR DE "Neurosis" OR DE "Paraphilias" OR DE "Personality Disorders" OR DE "Psychosis" OR DE "Serious Mental Illness" OR DE "Sleep Wake Disorders" OR DE "Somatoform Disorders" OR DE "Stress and Trauma Related Disorders" OR DE "Substance Related and Addictive Disorders" OR DE "Thought Disturbances" |
| --- | --- |
| 2 | (mental or psychiatric) n3 (illness or disorder* or disease*) |
| 3 | anxiety or depression or "mental health" |
| 4 | 1 or 2 or 3 |
| 5 | DE "Asians" OR DE "Chinese Cultural Groups" OR DE "Japanese Cultural Groups" OR DE "Korean Cultural Groups" OR DE "South Asian Cultural Groups" OR DE "Southeast Asian Cultural Groups" OR DE "Vietnamese Cultural Groups" OR DE "Vietnamese Cultural Groups" OR DE "Korean Cultural Groups" OR DE "Japanese Cultural Groups" OR DE "Chinese Cultural Groups" OR DE "Southeast Asian Cultural Groups" |
| 6 | East Asian or Korean or Hmong or Thai* or Lao* or Japan* or Vietnamese or Filipino or Chinese or Cambodian |
| 7 | 5 or 6 |
| 8 | 4 and 7 |
| 9 | DE "Health Attitudes" OR DE "Mental Illness (Attitudes Toward)" OR DE "Physical Illness (Attitudes Toward)" OR DE "Public Health Attitudes" OR DE "Vaccination Attitudes" OR DE "Racial and Ethnic Attitudes" OR DE "Antiracism" OR DE "AntiSemitism" OR DE "Color Blind Racial Attitudes" OR DE "Ethnocentrism" OR DE "Racism" OR DE "Preventive Mental Health Services" OR DE "Mental Health Disparities" OR DE "Health Disparities" OR DE "Mental Health Disparities" OR DE "Health Care Utilization" OR DE "Utilization Reviews" OR DE "Client Attitudes" OR DE "Client Satisfaction" OR DE "Treatment Barriers" OR DE "Treatment Compliance" |
| 10 | DE "Health Care Access" |
| 11 | DE "Treatment Dropouts" |
| 12 | DE "Treatment Refusal" |
| 13 | DE "Quality of Services" OR DE "Quality of Care" |
| 14 | experience* or outcome* or quality or satisfaction or attitude* or preference* or participation or engagement |
| 15 | DE "Narratives" |
| 16 | DE "Patient Centered Care" |
| 17 | DE "Interviews" OR DE "Cognitive Interview" OR DE "Focus Group Interview" OR DE "Intake Interview" OR DE "Interview Schedules" OR DE "Job Applicant Interviews" OR DE "Psychodiagnostic Interview" OR DE "Semi-Structured Interview" |
| 18 | DE "Biography" OR DE "Autobiography" |
| 19 | narration or "narrative medicine" or "patient-centered care" or interview or biography or autobiography or "personal narrative*" or "lived experience" or "patient narrative*" or "patient story" |
| 20 | 9 or 10 or 11 or 12 or 13 or 14 or 15 or 16 or 17 or 18 or 19 |
| 21 | adolescent* or "young adult*" or teen* |
| 22 | 8 and 20 and 21 |

Database(s): **CINAHL Complete** 1937– May 2023

| 1 | (MH "Mental Disorders+") |
| --- | --- |
| 2 | (mental or psychiatric) n3 (illness or disorder* or disease*) |
| 3 | anxiety or depression or "mental health" |
| 4 | 1 or 2 or 3 |
| 5 | (MH "Asians+") |
| 6 | (MH "China/EH") |
| 7 | (MH "Japan/EH") |
| 8 | (MH "Korea/EH") |
| 9 | (MH "Cambodia/EH") |
| 10 | (MH "Hmong/EH") |
| 11 | (MH "Laos/EH") |
| 12 | (MH "Philippines/EH") |
| 13 | (MH "Thailand/EH") |
| 14 | (MH "Vietnam/EH") |
| 15 | "East Asian" or Korean or Hmong or Thai* or Lao* or Japan* or Vietnamese or Filipino or Chinese or Cambodian |
| 16 | 5 or 6 or 7 or 8 or 9 or 10 or 11 or 12 or 13 or 14 or 15 |
| 17 | 4 and 16 |
| 18 | (MH "Attitude") OR (MH "Attitude of Health Personnel+") OR (MH "Attitude to Change") OR (MH "Attitude to Medical Treatment") OR (MH "Attitude to Disability") OR (MH "Attitude to Health") OR (MH "Health Beliefs") OR (MH "Patient Compliance+") OR (MH "Patient Satisfaction+") OR (MH "Attitude to Illness+") OR (MH "Treatment Refusal+") OR (MH "Family Attitudes+") OR (MH "Parental Attitudes+") OR (MH "Patient Attitudes") OR (MH "Personal Satisfaction+") OR (MH "Caregiver Attitudes") OR (MH "Social Attitudes") OR (MH "Student Attitudes+") |
| 19 | (MH "Patient Dropouts") |
| 20 | (MH "Quality of Health Care+") OR (MH "Outcomes (Health Care)+") OR (MH "Healthcare Disparities") OR (MH "Health Services Accessibility+") |
| 21 | experience* or satisfaction or quality or outcome* or attitude* or preference* or participation or engagement |
| 22 | (MH "Narratives+") |
| 23 | (MH "Patient Centered Care") |
| 24 | (MH "Autobiographies") OR (MH "Biographies+") |
| 25 | narration or "narrative medicine" or "patient-centered care" or interview or biography or autobiography or "personal narrative*" or "lived experience" or "patient narrative*" or "patient story" |
| 26 | 18 or 19 or 20 or 20 or 21 or 22 or 23 or 24 or 25 |
| 27 | adolescent* or young adult* or teen* |
| 28 | 17 and 26 and 27 |

Database(s): **Sociology Collection** 1962– May 2023

(((mental OR psychiatric) NEAR/3 (illness OR disorder* OR disease*)) OR (anxiety OR depression OR "mental health")) AND ("East Asian") AND ((experience* OR outcome* OR quality OR satisfaction OR attitude* OR preference* OR participation OR engagement) OR (narration OR "narrative medicine" OR "patient-centered care" OR interview OR biography OR autobiography) OR ("personal narrative" OR "personal narratives") OR ("lived experience" OR "patient narrative*" OR "patient story")) AND (adolescent* OR young adult* OR teen*)
